# Clinical, Dermatoscopic, Histological and Molecular Prognostic and Predictive Factors of Metastatic Melanoma Response to Immunotherapy: A Systematic Review and Drug Class Meta-Analysis

**DOI:** 10.3390/jcm15062145

**Published:** 2026-03-11

**Authors:** Michail C. Papazoglou, Chrysostomos Avgeros, Eleni Sogka, Anestis Chrysostomidis, Georgios Karakinaris, Anastasios Boutis, Aimilios Lallas, Athanassios Kyrgidis

**Affiliations:** 1 School of Medicine, Faculty of Health Sciences, Aristotle University of Thessaloniki, 54 124 Thessaloniki, Greece; michalis.papazoglou@me.com; 2 Department of Medical Oncology, General Hospital of Thessaloniki “George Papanikolaou”, Exochi, 57 010 Thessaloniki, Greece; chrysavgeros@gmail.com (C.A.); elenisogka@gmail.com (E.S.); 3 Oral and Maxillofacial Surgery, Aristotle University of Thessaloniki, 54 124 Thessaloniki, Greece; achrisostomidis@gmail.com; 4 First Department of Dermatology, School of Medicine, Faculty of Health Sciences, Aristotle University, 54 643 Thessaloniki, Greece; 5 Laboratory of Oral & Maxillofacial Surgery, Dental School, Aristotle University of Thessaloniki, 54 124 Thessaloniki, Greece; karakinarisg@gmail.com; 6 Third Department of Clinical Oncology, Theagenio Cancer Hospital, 54 639 Thessaloniki, Greece; alboutis@gmail.com; 7 Second Dermatology Department, School of Health Sciences, Aristotle University of Thessaloniki, 54 643 Thessaloniki, Greece; emlallas@gmail.com; 8 Specialized Cancer Treatment and Reconstruction Center, Department of Oral and Maxillofacial Surgery, General Hospital of Thessaloniki “George Papanikolaou”, Exochi, 57 010 Thessaloniki, Greece; 9 Laboratory of Clinical Pharmacology, Medical School, Aristotle University of Thessaloniki, 54 124 Thessaloniki, Greece

**Keywords:** metastatic melanoma, immune checkpoint inhibitors, predictive biomarkers, overall survival, progression-free survival, systematic review, meta-analysis

## Abstract

**Introduction:** Immune checkpoint inhibitors (ICIs) have transformed the treatment of metastatic melanoma; however, predictive markers of therapeutic response remain poorly defined. This study systematically assesses clinical, histological, and molecular predictors associated with survival outcomes in melanoma patients treated with ICIs. **Methods:** Following the Preferred Reporting Items for Systematic Reviews and Meta-Analyses (PRISMA) and the Meta-Analysis of Observational Studies in Epidemiology (MOOSE) guidelines, a systematic search was conducted in MEDLINE, Web of Science, and the Cochrane Central Register of Controlled Trials (CENTRAL) for studies published between January 2018 and October 2025. Eligible studies reported associations between predictive factors and overall survival (OS) or progression-free survival (PFS) in adult melanoma patients receiving ICIs. Pooled hazard ratios (HRs) with corresponding 95% confidence intervals (CIs) from univariate (UVA) and multivariate analyses (MVA) were synthesized using random-effects meta-analyses. **Results:** Sex was not a consistent predictor (contradictory effects; PFS heterogeneity I^2^ ≈ 90%), whereas older age predicted worse OS (MVA continuous: HR 1.05, 95% CI 1.02–1.08; UVA ≥ 65 vs. <65: HR 1.70, 95% CI 1.36–2.12). Poor performance status, assessed using the Eastern Cooperative Oncology Group (ECOG) scale, strongly predicted inferior outcomes (ECOG ≥ 1 vs. 0: MVA OS HR 2.01, 95% CI 1.61–2.51; MVA PFS HR 1.49, 95% CI 1.18–1.88; ECOG ≥ 2 vs. <2: MVA OS HR 2.24, 95% CI 1.79–2.81). Elevated lactate dehydrogenase (LDH) was consistently associated with poorer survival (MVA OS HR 1.71, 95% CI 1.53–1.91; MVA PFS HR 1.61, 95% CI 1.41–1.85), whereas body mass index (BMI) > 25 kg/m^2^ was associated with improved OS (HR 0.82, 95% CI 0.68–0.98). Higher disease burden predicted worse prognosis (Stage IV vs. III: MVA OS HR 1.57, 95% CI 1.16–2.13; >2 metastatic sites vs. ≤2: MVA OS HR 2.38, 95% CI 1.40–4.07; brain metastases: MVA OS HR 1.69, 95% CI 1.30–2.20; MVA PFS HR 1.52, 95% CI 1.00–2.33). Histologic and molecular factors showed prognostic value: ulceration worsened OS (UVA HR 2.08, 95% CI 1.25–3.44) and PFS (UVA HR 2.97, 95% CI 1.39–6.32); acral subtype had poorer OS than cutaneous melanoma (MVA HR 2.99, 95% CI 1.63–5.48); high tumor mutational burden (TMB) improved PFS (UVA HR 0.47, 95% CI 0.33–0.70); and cutaneous immune-related adverse events (irAEs) were associated with favorable outcomes (skin disorders: UVA OS HR 0.26, 95% CI 0.14–0.47; UVA PFS HR 0.50, 95% CI 0.34–0.74). In contrast, detectable circulating tumor DNA (ctDNA) predicted markedly worse PFS (MVA HR 4.72, 95% CI 2.31–9.65) and a non-significant trend toward worse OS (MVA HR 3.34, 95% CI 0.96–11.67). Liver metastases and programmed death-ligand 1 (PD-L1) expression were not significantly associated with survival. **Discussion:** This meta-analysis synthesizes evidence on clinicopathologic, laboratory, and histopathologic predictors of immunotherapy outcomes in metastatic melanoma. Performance status, age, LDH, BMI, and metastatic burden consistently correlated with prognosis, while ulceration, disease stage, and TMB emerged as key histologic determinants. Conversely, PD-L1 and gender showed no consistent predictive value, whereas cutaneous immune-related adverse events and ctDNA reflected favorable and poor outcomes, respectively. These findings highlight the multifactorial nature of immunotherapy response and support the further development of integrated prognostic models to refine patient stratification and optimize treatment outcomes.

## 1. Introduction

Metastatic melanoma is a highly aggressive form of cancer with historically poor outcomes, and its incidence continues to rise. According to GLOBOCAN 2022 [1], melanoma ranked as the 17th most common cancer worldwide, with approximately 331,722 new cases and 58,667 deaths. U.S.-based estimates for 2024 project around 200,340 new melanoma diagnoses (100,640 invasive) with 7990 deaths [2]. Europe reports over 150,000 new melanoma cases annually, resulting in more than 25,000 deaths [3]. The lifetime cumulative risk of developing melanoma in Europe is approximately 2.06% [4].

The advent of novel therapeutic approaches, particularly immune checkpoint inhibitors (ICIs) and targeted therapies, has significantly transformed the treatment landscape and improved survival in patients with advanced disease. Immune checkpoints are regulatory proteins found on T cells that help to maintain immune homeostasis and prevent autoimmune reactions. By inhibiting these checkpoints, ICIs restore T-cell activity and enable an immune-mediated attack on tumor cells [5].

The first ICI to receive approval from the U.S. Food and Drug Administration (FDA) was ipilimumab in 2011. Ipilimumab targets cytotoxic T-lymphocyte-associated antigen 4 (CTLA-4), a receptor that downregulates immune responses. Blocking CTLA-4 enhances T-cell activation and promotes antitumor immunity. Clinical trials showed an increase in median survival from 6.4 months to 10 months in melanoma patients treated with ipilimumab, compared to those receiving a vaccine or dacarbazine. A plateau in survival curves after two to three years suggested durable responses in a subset of patients. However, the overall response rate was below 20%, and serious immune-related adverse events (grade 3–4) occurred in approximately 15–45% of patients [6].

Subsequent trials comparing ipilimumab to anti-programmed death-1 (PD-1) antibodies—such as nivolumab and pembrolizumab—yielded more favorable outcomes. Anti-PD-1 therapies disrupt the inhibitory signals that dampen effector T-cell activity by blocking their interaction with PD-L1/2, allowing T cells to remain functional and capable of killing tumor cells. These therapies demonstrated objective response rates of 20–30% as monotherapy, and up to 40–50% when combined with CTLA-4 blockade. Notably, grade 3–4 toxicities were reduced to 11–16% with anti-PD-1 monotherapy, though remained high (around 40%) in combination regimens due to the inclusion of anti-CTLA-4 agents [7,8,9].

Despite improved response rates with ICIs—especially anti-PD-1 agents—long-lasting benefits are limited to a fraction of patients. Furthermore, the risk of serious toxicities, including gastrointestinal, hepatic, and dermatological side effects, remains considerable, particularly with combination therapy. Consequently, predictors are urgently needed to identify patients who are most likely to benefit from ICIs, while avoiding unnecessary toxicity and financial costs in non-responders. Several predictors have been investigated, ranging from serum lactate dehydrogenase (LDH) levels to tumor mutation burden (TMB), PD-L1 expression, and tumor-infiltrating lymphocytes (TILs) [10,11]. Except for LDH, none of these markers have yet been adopted into routine clinical practice. This systematic review aims to comprehensively summarize the current evidence on, demographic, blood-based, tumor-derived, and any other clinically meaningful predictors associated with treatment response, progression-free survival, and overall survival in patients with metastatic melanoma receiving ICI therapy.

## 2. Materials and Methods

### 2.1. Guidelines Followed

**Figure 1:**
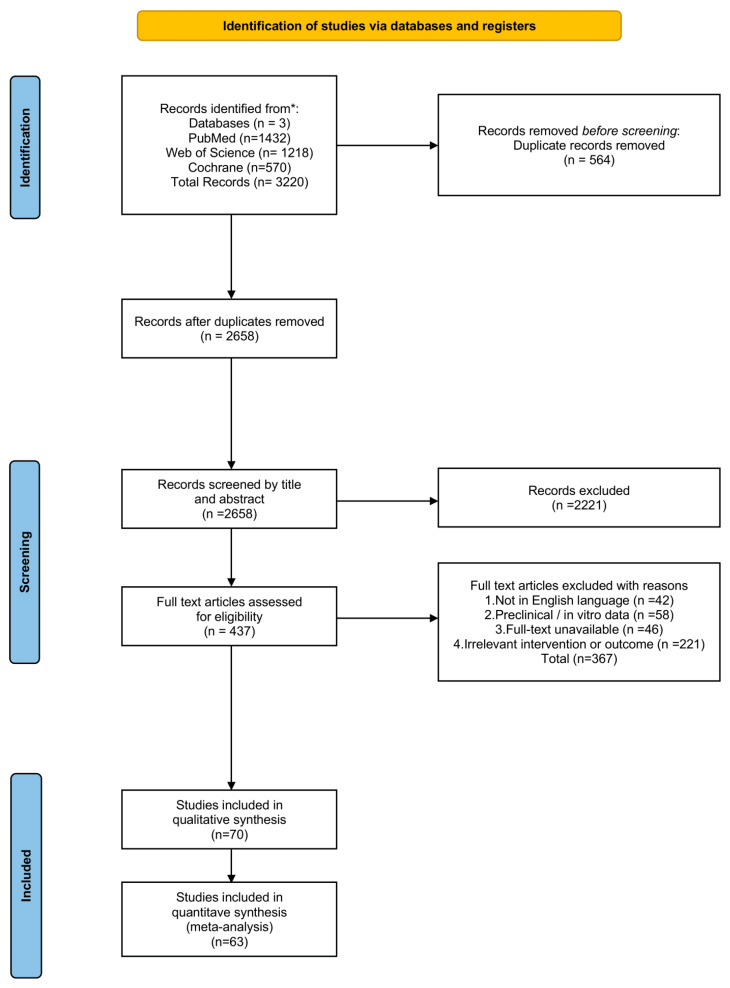
Flow chart diagram. * 1 January 2018 to 31 October 2025.

This systematic review was conducted and reported in accordance with the Preferred Reporting Items for Systematic Reviews and Meta-Analyses (PRISMA) 2020 statement [12]. The completed PRISMA 2020 checklist is provided in Supplementary Table S5. Figure 1 presents the PRISMA flow diagram. The study was registered in PROSPERO (International Prospective Register of Systematic Reviews; registration number: CRD420251269550).

### 2.2. Search Strategy and Eligibility Criteria

The present systematic review and meta-analysis was conducted in accordance with the PRISMA 2020 guidelines. A comprehensive and systematic search strategy was employed to identify relevant studies in the electronic databases MEDLINE (via PubMed), Web of Science, and the Cochrane Central Register of Controlled Trials (CENTRAL), covering publications from 1 January 2018 to 31 October 2025. The search was independently performed by two investigators (C.A. and M.P.). Discrepancies during the study selection process were resolved through consensus with a third reviewer (A.K.), ensuring methodological rigor and minimizing the risk of selection bias.

The search strategy combined keywords and Medical Subject Headings (MeSH) terms including “melanoma,” “immune checkpoint inhibitors,” “ICI,” “PD-1 inhibitor (PD-1i),” and “CTLA-4 inhibitor (CTLA-4i).” Study design-specific filters were applied to enhance search specificity. The full search strategies for each database are provided in Supplementary Tables S1–S3.

Eligible studies were required to investigate clinical, histopathological, or molecular predictive factors associated with therapeutic outcomes in patients with melanoma receiving immunotherapy. Clinical parameters of interest included patient age, sex, and anatomical location of the primary tumor. Histopathological features encompassed histologic subtype, Breslow thickness, ulceration, mitotic index, evidence of regression, and sentinel lymph node (SLN) status. Molecular biomarkers included BRAF and NRAS mutational profiles and gene expression signatures linked to treatment response. For the purposes of this analysis, non-cutaneous melanoma was defined as acral and mucosal melanoma, while uveal melanoma was excluded unless explicitly reported.

Studies were considered eligible if they met the following criteria: (i) publication date between 1 January 2018, and 31 October 2025; (ii) a study population comprising adult patients (aged 18 years or older) diagnosed with melanoma and treated with a programmed death-1/programmed death-ligand 1 (PD-(L)1) inhibitor, a cytotoxic T-lymphocyte-associated antigen 4 (CTLA-4) inhibitor, or a combination of these agents; (iii) availability of hazard ratios (HRs) with corresponding 95% confidence intervals (CIs) for survival outcomes, including progression-free survival (PFS), overall survival (OS), or both; (iv) study designs including randomized controlled trials, prospective or retrospective cohort studies, and cross-sectional studies. No minimum sample size threshold was applied. Studies were excluded if they were not published in English, involved only preclinical or in vitro data, fell outside the specified publication date range, or did not report on either the relevant therapeutic interventions (immune checkpoint inhibitors) or the primary outcomes of interest (OS, PFS, or HRs).

Potential overlap of patient cohorts was carefully assessed during study selection and data extraction, particularly for studies originating from the same institutions, registries, or overlapping recruitment periods. When overlap was suspected, the study with the largest sample size, longest follow-up, or most comprehensive reporting was preferentially included in the quantitative synthesis, while overlapping datasets were excluded to avoid double-counting. Importantly, studies deriving from overlapping patient cohorts were allowed to contribute to the meta-analysis only when they addressed different predictors or different clinical endpoints; however, for any given predictor–endpoint combination (e.g., overall survival or progression-free survival), overlapping patient populations were not included simultaneously in the quantitative synthesis.

### 2.3. Data Extraction

Two independent researchers extracted data from eligible studies using a standardized data extraction form. The following parameters were recorded: (i) first author; (ii) year of publication; (iii) country in which the study was conducted; (iv) study design; (v) duration of follow-up for cohort studies; (vi) total number of patients included and the number who developed distant metastasis (DM); (vii) predictors of survival and treatment outcomes in metastatic melanoma; (viii) reported outcomes, including distant metastasis-free survival (DMFS) and time to distant metastasis (TTDM).

The primary outcome was the comparison between patients who exhibited differential responses to immune checkpoint inhibitor (ICI) therapy during follow-up. The predictive role of reported clinicopathological and molecular factors on survival outcomes—reported as overall survival (OS) or progression-free survival (PFS)—was analyzed. Subgroup analyses were conducted according to American Joint Committee on Cancer (AJCC) stage (patients with Stage I–II disease or sentinel lymph node-negative status) and study design (cohort or case–control studies), when feasible.

### 2.4. Risk of Bias and Study Quality Assessment

The methodological quality and risk of bias of the included observational studies were rigorously evaluated using the Newcastle–Ottawa Scale (NOS), a well-established tool for appraising non-randomized studies in meta-analyses. The NOS assesses studies across three domains: selection of study participants (up to 4 points), comparability of groups based on study design or analysis (up to 2 points), and ascertainment of outcomes and adequacy of follow-up (up to 3 points), with a maximum total score of 9. Independent assessments were performed by three reviewers. Disagreements between reviewers regarding the quality assessment were thoroughly discussed and reconciled through a standardized consensus process, ensuring consistency with the predefined methodological criteria. Studies scoring between 7 and 9 were classified as having a low risk of bias, scores of 5–6 were considered indicative of moderate risk, while scores below 5 suggested a high risk of bias. This approach ensured a clear and consistent assessment of study validity, enhancing the reliability of the meta-analysis findings. A detailed summary of NOS ratings for each included study is available in Supplementary Table S4.

### 2.5. Statistical Analysis

Meta-analytical techniques were employed to synthesize evidence on the association between selected clinical and pathological predictors with the outcomes of overall survival (OS) and progression-free survival (PFS). Hazard ratios (HRs) along with their corresponding 95% confidence intervals (CIs) were extracted directly from the primary studies. When both univariate (UVA) and multivariate (MVA) estimates were available, they were analyzed separately to preserve the methodological integrity of the reported models. Univariate HRs were typically derived from Kaplan–Meier survival curves or log-rank tests, whereas multivariate estimates originated from Cox proportional hazards models adjusted for study-specific covariates.

Pooled analyses were conducted using random-effects models based on the DerSimonian–Laird estimator, incorporating the Hartung–Knapp adjustment to improve the reliability of confidence intervals, particularly in the presence of between-study variance. Logarithmic transformation of HRs was applied prior to analysis, and standard errors were computed from the reported CIs using established formulas.

Heterogeneity across studies was assessed using the I^2^ statistic, with thresholds of <30%, 30–60%, and >60% interpreted as low, moderate, and substantial heterogeneity, respectively. Additional indices, including the between-study variance (τ^2^) and Cochran’s Q statistic, were also calculated to provide a comprehensive evaluation of heterogeneity.

Forest plots were constructed for each predictor to illustrate the effect estimates from individual studies alongside the overall pooled estimate. Sensitivity analyses were performed using a leave-one-out approach to determine the influence of individual studies on the meta-analytic estimates; a change exceeding 0.15 in the pooled HR was considered indicative of a significant influence.

All analyses were performed using R (version 4.4.0) within the RStudio environment (version 2025.05.1), employing the packages meta, dplyr, and readxl. Statistical significance was set at a two-sided *p*-value threshold of <0.05.

## 3. Results

Sixty-nine studies satisfied the inclusion criteria and were included in this systematic review (Table 1). We included 63 studies in the quantitative syntheses [13,14,15,16,17,18,19,20,21,22,23,24,25,26,27,28,29,30,31,32,33,34,35,36,37,38,39,40,41,42,43,44,45,46,47,48,49,50,51,52,53,54,55,56,57,58,59,60,61,62,63,64,65,66,67,68,69,70,71,72,73,74,75,76]. The results are presented per predictor studied below. Sensitivity analyses excluding CTLA-4 monotherapy cohorts confirmed that the pooled estimates remained robust across major predictors.

### 3.1. Demographic Predictors

#### 3.1.1. Gender

Despite its evaluation across 63 identified studies, gender did not emerge as a consistent predictor of survival outcomes [1]. Among the seven studies reporting statistically significant associations, the direction of effect was highly contradictory. In multivariate analyses for overall survival (OS), estimates ranged drastically from a protective effect for females (HR = 0.74) to an extreme hazard ratio (HR = 60.4) in a small cohort, precluding meaningful synthesis. Similarly, analyses of progression-free survival (PFS) demonstrated significant but opposing associations (HR = 1.22 favoring males vs. HR = 0.34 favoring females), resulting in prohibitive heterogeneity (I^2^ ≈ 90%). Consequently, due to the conflicting directions of effect and marked statistical variance, a quantitative meta-analysis was not feasible.

#### 3.1.2. Age

##### Age (Continuous)

MVA

**Figure 2:**
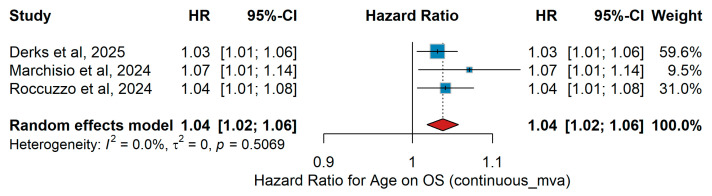
Forest plot for predictive factors of metastatic melanoma response to immunotherapy. Age was analyzed as a continuous variable. Increasing age was significantly associated with worse overall survival (OS).

A total of three studies were included in the analysis evaluating age as a continuous variable for OS. Through the four studies, patient populations varied in size and clinical setting. The pooled HR was 1.05 (95% CI 1.02–1.08, *p* < 0.001), with moderate heterogeneity (I^2^ = 48.7%, *p* = 0.119), indicating that overall survival decreased with increasing age. The corresponding forest plot is presented in Figure 2.

##### Sensitivity Analysis

(ΜVA OS)

Leave-one-out sensitivity analysis (Figure 2) confirmed the robustness of the multivariate association between age and overall survival. The pooled hazard ratio remained highly stable (range: 1.04–1.07) with negligible heterogeneity (I^2^ < 1%), indicating that the prognostic impact of age is consistent across independent cohorts and not driven by any single study.

##### Age (Dichotomous ≥80 vs. <80)

MVA

**Figure 3:**
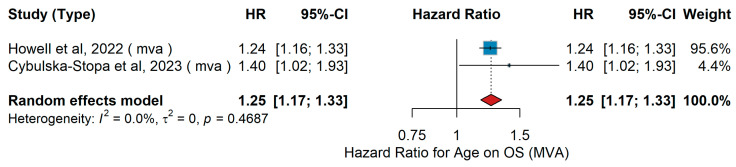
Forest plot for predictive factors of metastatic melanoma response to immunotherapy (Multivariate Analyses). Age as dichotomous variable. Older age was significantly associated with worse overall survival (OS).

A total of 1435 older patients with cutaneous melanoma (aged ≥80 years; mean age 76.6 years) were analyzed in the Howell et al. [53] U.S. SEER-Medicare cohort. The Cybulska-Stopa et al. [36] Polish multicenter study included 1037 patients with inoperable or metastatic melanoma, of whom 455 received pembrolizumab and 582 received nivolumab as first-line PD-1 therapy. Based on both studies, younger patients demonstrated better survival outcomes compared to older patients, particularly when treated with PD-1-based immunotherapy, which was associated with prolonged overall survival relative to CTLA-4 inhibitors. The pooled hazard ratio derived from the multivariable analyses is illustrated in Figure 3.

##### Age (Dichotomous ≥ 65 vs. <65)

UVA

**Figure 4:**
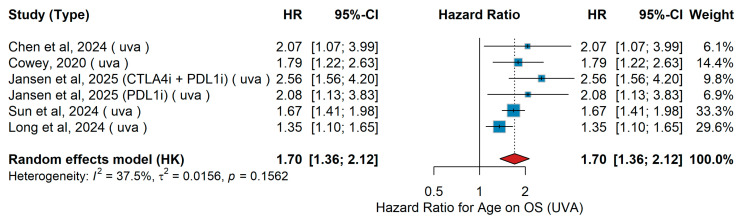
Forest plot for predictive factors of metastatic melanoma response to immunotherapy (Univariate Analysis). Age as dichotomous variable. Older age was significantly associated with worse overall survival (OS).

A total of four studies were included in the analysis evaluating age as a dichotomous variable (≥65 vs. <65 years) for overall survival (OS). These studies’ patient populations differed in size, ethnicity, and treatment setting. Chen et al. [32] retrospectively analyzed 53 Chinese patients with metastatic melanoma treated with either immune checkpoint inhibitors (PD-1 ± CTLA-4) or other systemic therapies. Cowey et al. [60] included 224 U.S. patients with BRAF-mutant advanced melanoma, treated in a community oncology network with either anti-PD-1 monotherapy (36.2%) or BRAF/MEK inhibitors (63.8%). Sun et al. [19] retrospectively evaluated 140 Spanish patients with advanced melanoma, including 82 with BRAF mutations, treated with targeted therapy (ΤΤ) (BRAF/MEKi, n = 50) or immunotherapy (anti-PD-1 or nivolumab + ipilimumab, n = 90). In addition, Jansen et al. [14] reported on 277 patients with metastatic melanoma receiving PD-1 or CTLA-4 + PD-1 inhibitors, with HRs for age ranging from 2.08 (95% CI 1.13–3.84, *p* = 0.02) for PD-1 monotherapy to 2.56 (95% CI 1.56–4.20, *p* = 0.001) for combination therapy. The pooled hazard ratio (HR) was 1.70 (95% CI: 1.36–2.12, *p* < 0.001), indicating that older age was significantly associated with worse survival. Heterogeneity across studies was moderate (I^2^ = 37.5%, *p* = 0.1562). These findings are illustrated in Figure 4.

Sensitivity Analysis (UVA OS)

Leave-one-out sensitivity analysis confirmed the prognostic stability of age for overall survival. The pooled hazard ratio remained consistent across all iterations (range: 1.35–2.56), indicating that the association is robust and not driven by any single study or therapeutic context.

### 3.2. Clinical Predictors

#### 3.2.1. ECOG

##### ECOG (≥1 vs. 0)

MVA OS

**Figure 5:**
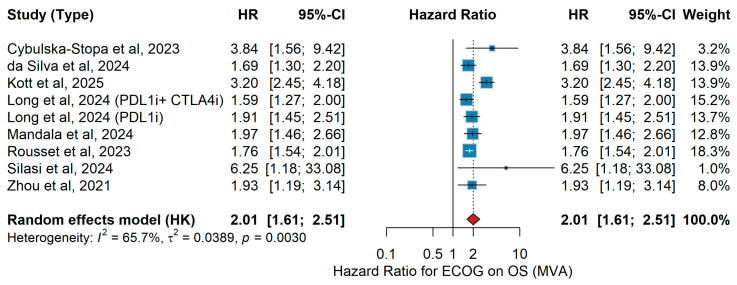
Forest plot of multivariate analysis for overall survival (OS) in metastatic melanoma. ECOG as a dichotomous variable. Poor performance status (higher ECOG) was significantly associated with worse overall survival (OS).

In multivariate analyses (MVA) of studies investigating patients with advanced or metastatic melanoma treated with immune checkpoint inhibitors (ICIs), performance status consistently emerged as an independent predictor of overall survival (OS). Most studies demonstrated significantly poorer outcomes for patients with an ECOG performance status ≥ 1 compared to ECOG 0. In the pooled meta-analysis, an ECOG ≥ 1 was associated with more than a twofold increased risk of death compared to ECOG 0 (pooled HR = 2.01, 95% CI 1.61–2.51, *p* < 0.001), with moderate between-study heterogeneity (I^2^ = 65.7%, *p* = 0.0030). The corresponding forest plot is presented in Figure 5.

Sensitivity analysis

Leave-one-out sensitivity analysis confirmed the prognostic stability of ECOG performance status (≥1 vs. 0) for overall survival, with pooled hazard ratios remaining consistent across iterations (range: 1.78–2.10), indicating a robust and study-independent association.

UVA OS

**Figure 6:**
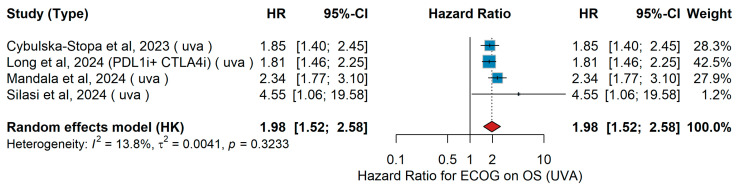
Forest plot of univariate analysis for overall survival (OS) in metastatic melanoma. ECOG as a dichotomous variable. Poor performance status (higher ECOG) was significantly associated with worse overall survival (OS).

A total of four studies were included in the univariate analysis (UVA) investigating the association between ECOG performance status and overall survival (OS). Cybulska-Stopa et al. [36] analyzed 1037 patients with advanced or metastatic melanoma treated with first-line anti-PD-1 therapy. Long et al. [44] conducted a pooled analysis of six CheckMate trials including 1375 patients treated with nivolumab plus ipilimumab or nivolumab monotherapy. Mandalà et al. [16] evaluated 376 patients with melanoma brain metastases treated with combined nivolumab and ipilimumab. Silasi et al. [42] presented real-world outcomes from 53 Stage IV melanoma patients in Romania receiving nivolumab plus ipilimumab. The pooled random-effects meta-analysis demonstrated that ECOG performance status ≥ 1 versus 0 was significantly associated with inferior overall survival, with a combined hazard ratio of 1.98 (95% CI 1.52–2.58) (*p* = 0.00387). Between-study heterogeneity was low (I^2^ = 13.8%, *p* = 0.3233). Figure 6 displays the forest plot summarizing the association between ECOG performance status and overall survival in the univariable analysis.

MVA PFS

**Figure 7:**
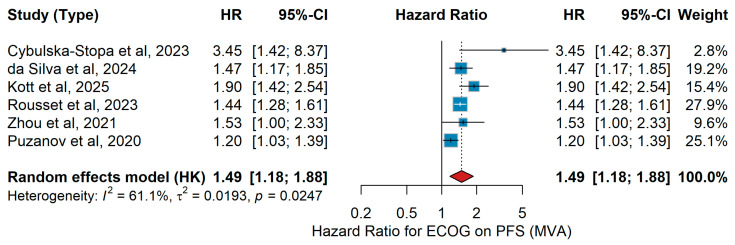
Forest plot of multivariate analysis for progression-free survival (PFS) in metastatic melanoma. ECOG as a dichotomous variable. Poor performance status (higher ECOG) was significantly associated with worse progression-free survival (PFS).

In multivariable analyses (MVAs), poorer ECOG performance status was consistently associated with inferior progression-free survival (PFS) across most studies. Cybulska-Stopa et al. [36] reported significantly shorter PFS for patients with poorer ECOG status, while da Silva et al. [18], Kott et al. [15], Rousset et al. [25], Zhou et al. [21], and Puzanov et al. [45] (HR 1.20, 95% CI 1.03–1.39) all demonstrated a detrimental effect of poor ECOG performance on PFS. The pooled random-effects meta-analysis confirmed that worse ECOG performance status (primarily ECOG ≥ 1 vs. 0) was associated with shorter PFS (HR 1.49, 95% CI 1.18–1.88), with moderate heterogeneity (I^2^ = 61.1%, *p* = 0.0247), as shown in Figure 7.

Sensitivity analysis

Leave-one-out analysis did not significantly alter the pooled estimate (HR range: 1.40 to 1.53). Across datasets, the effect of ECOG consistently ranged from HR 1.2 to 1.9, fully concordant with our pooled estimate. Overall, the sensitivity analyses show that although large datasets (e.g., registry-based studies) can modestly shift pooled HRs, the association between ECOG performance status and PFS remains consistent and generalizable across diverse populations and therapeutic settings [18,42,44].

UVA PFS

**Figure 8:**
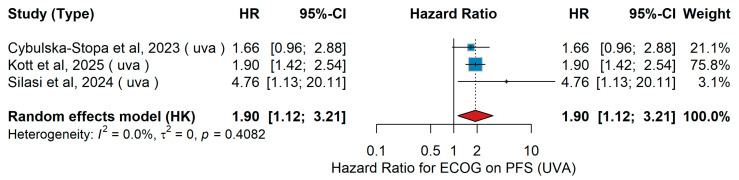
Forest plot of univariate analysis for progression-free survival (PFS) in metastatic melanoma. ECOG as a dichotomous variable. Poor performance status (higher ECOG) was significantly associated with worse progression-free survival (PFS).

In the univariate analysis, Cybulska-Stopa et al. [36] evaluated 1037 patients with unresectable or metastatic melanoma treated in the first line with anti-PD-1 antibodies (nivolumab or pembrolizumab). Kott et al. [15] included patients receiving systemic therapy in a real-world setting and reported ECOG as an independent predictive factor for PFS (HR 1.90, 95% CI 1.42–2.54, *p* < 0.001). Silasi et al. [42] retrospectively analyzed 53 patients with Stage IV melanoma treated with combined nivolumab and ipilimumab across two Romanian centers. The pooled analysis in the random-effects model comparing ECOG ≥ 1 versus ECOG 0 showed a hazard ratio of 1.90 (95% CI 1.12–3.21, *p* = 0.016). Heterogeneity was low (I^2^ = 0%, *p* = 0.4082). These results are illustrated in Figure 8.

##### ECOG (≥2 vs. <2)

MVA OS

**Figure 9:**
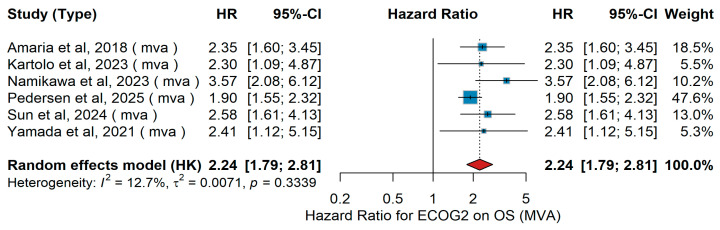
Forest plot of multivariate analysis for overall survival (OS) in metastatic melanoma. ECOG as a dichotomous variable. ECOG ≥ 2 was significantly associated with worse overall survival (OS).

Subsequently, we focused on the comparison of ECOG categories, primarily evaluating the impact of ≥2 versus <2. Across six eligible studies, multivariate analyses assessed the impact of ECOG performance status on overall survival (OS). Populations and treatments varied: Amaria et al. [31] enrolled patients with high-risk, surgically resectable Stage III/oligometastatic Stage IV BRAF V600 melanoma who received neoadjuvant + adjuvant dabrafenib/trametinib versus standard of care. Kartolo et al. [26] analyzed advanced/metastatic melanoma patients who had permanently discontinued PD-1 ± CTLA-4 therapy in routine practice. Namikawa et al. [56] reported a Japanese multicenter real-world cohort of BRAF V600 mutant advanced melanoma first-line-treated with BRAF/MEK inhibitors, anti-PD-1, or nivolumab + ipilimumab. Pedersen et al. [17] included a nationwide Danish cohort of patients with melanoma brain metastases (±leptomeningeal disease) treated with immune checkpoint inhibitors or BRAF/MEK inhibitors alongside local therapies. Sun et al. [19] described a single-center decade-long series of BRAF-mutated metastatic melanoma first-line-treated with immunotherapy or BRAF/MEK inhibitors. Yamada et al. [62] evaluated gastrointestinal immune-related adverse events in patients receiving immune checkpoint inhibitors across solid tumors, including melanoma. When pooled in a random-effects meta-analysis, ECOG ≥ 2 was associated with significantly worse overall survival (pooled HR = 2.24, 95% CI 1.79–2.81), with low heterogeneity across studies (I^2^ = 12.7%, *p* = 0.3339). Figure 9 presents the corresponding forest plot of the pooled multivariable estimates.

Sensitivity analysis

For ECOG ≥ 2 vs. <2, leave-one-out sensitivity analyses yielded HRs ranging from 2.08 to 2.60. Despite moderate heterogeneity, all estimates remained >2, indicating a robust association with poorer overall survival. This is consistent with prior evidence identifying baseline performance status as a strong prognostic factor, supporting ECOG as a key stratification variable in clinical practice and trial design [31].

UVA OS

**Figure 10:**
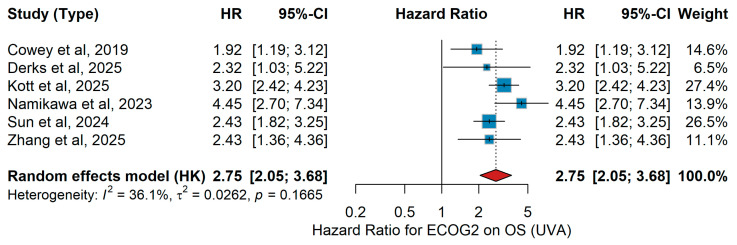
Forest plot of univariate analysis for overall survival (OS) in metastatic melanoma. ECOG as a dichotomous variable. ECOG ≥ 2 was significantly associated with worse overall survival (OS).

**Figure 11:**
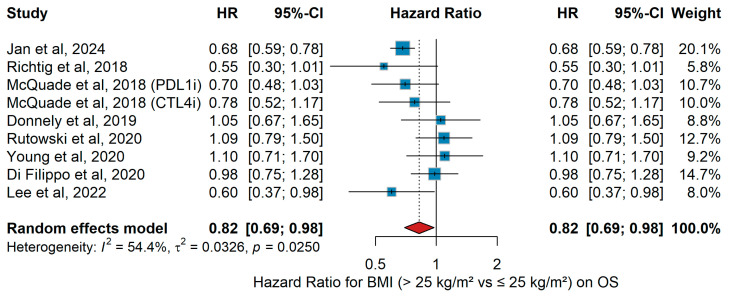
Forest plot of multivariate and univariate analysis for overall survival (OS) in metastatic melanoma. BMI as a dichotomous variable. Higher BMI was significantly associated with improved overall survival (OS).

In univariate analyses, ECOG performance status 2 versus ECOG 0–1 was consistently associated with worse overall survival (OS) across studies included in the meta-analysis (Figure 10). The following effect estimates were used: Cowey et al. [61], Derks et al. [24], Kott et al. [15], Namikawa et al. [56]; Sun et al. [19]; Zhang et al. [20]. The random-effects (HK) pooled estimate demonstrated a significant association between ECOG ≥ 2 versus ECOG < 2 and overall survival: HR = 2.75 (95% CI 2.05–3.68). Between-study heterogeneity was substantial (I^2^ = 36.1%, *p* = 0.1665), with most individual estimates favoring improved survival among patients with BMI > 25 kg/m^2^ (Figure 11).

Sensitivity analysis

For ECOG ≥ 2 and overall survival, HRs ranged from 1.94 to 2.60 across sensitivity analyses, consistently indicating increased mortality risk. The association remained positive in all scenarios, with no single study materially altering the pooled effect.

#### 3.2.2. BMI (>25 kg/m^2^ vs. ≤25 kg/m^2^)

UVA & MVA

The aim of the meta-analysis by Roccuzzo et al. [57] was to systematically review and quantitatively synthesize data from clinical studies [15,50,67,69,71,72,73,74,76] evaluating the relationship between overweight/obesity (generally defined as BMI > 25 kg/m^2^) and survival outcomes in patients with advanced melanoma treated with immune-checkpoint inhibitors (ICIs) such as anti-PD-1 and anti-CTLA-4 agent. After screening 1070 articles and including 18 for the systematic review, 7 studies met criteria for inclusion in quantitative meta-analysis. The pooled hazard ratio (HR) for overall survival (OS) comparing overweight/obese versus non-overweight patients was 0.87 (95% CI: 0.74–1.03). Although these point estimates suggest a modest advantage in overweight/obese patients, neither result reached conventional statistical significance. In an updated mixed-effects meta-analysis including two additional studies [50,54] along with those from Roccuzzo et al. [57], we re-examined the association between body mass index (BMI) and overall survival (OS) in patients with advanced melanoma treated with immune-checkpoint inhibitors. The pooled hazard ratio (HR) across nine studies was 0.82 (95% CI 0.69–0.98, *p* < 0.05), suggesting a modest but statistically significant survival advantage for overweight/obese patients (BMI > 25 kg/m^2^). Between-study heterogeneity was moderate (I^2^ = 54.4%, *p* = 0.025), with most individual estimates favoring improved survival among patients with BMI > 25 kg/m^2^ (Figure 11). While this updated analysis strengthens the evidence for a possible “obesity-paradox” effect on OS, the wide variability and remaining heterogeneity indicate that BMI alone still cannot be considered a robust or independent prognostic biomarker for melanoma patients receiving immunotherapy.

Sensitivity Analysis (OS)

Sensitivity analyses for BMI and overall survival showed consistent results across studies, indicating a stable pooled association not driven by any single study.

#### 3.2.3. LDH

LDH (high vs. low)
MVA OS

**Figure 12:**
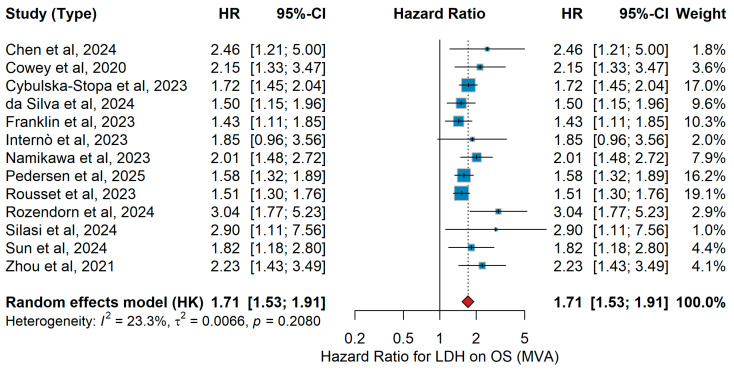
Forest plot of multivariate analysis for overall survival (OS) in metastatic melanoma. LDH as a dichotomous variable. Elevated LDH levels were significantly associated with worse overall survival (OS).

In the multivariable analysis (MVA), heterogeneous patient populations and systemic therapies were evaluated. Ascierto et al. [23] analyzed 71 patients with metastatic melanoma treated with anti-PD-1 agents, reporting inferior OS in patients with elevated LDH. In a Chinese cohort of 53 patients predominantly with acral and mucosal subtypes, Chen et al. [32] observed poorer OS under PD-1 blockade in those with increased LDH. Cowey et al. [60] investigated 224 BRAF-mutant patients receiving first-line anti-PD-1 or BRAF/MEK inhibitors, demonstrating worse survival with elevated LDH. Cybulska-Stopa et al. [36] reported on 1037 patients treated with pembrolizumab or nivolumab in the first line, showing significantly inferior OS for those with high LDH. In a large international cohort of patients with liver metastases, da Silva et al. [18] confirmed the negative predictive effect of elevated LDH regardless of anti-PD-1 or PD-1 plus CTLA-4 therapy. Franklin et al. [38], in the ADOREG registry of 1704 patients treated with ICIs or ΤΤ, identified high LDH as an independent adverse predictive factor. Internò et al. [39] evaluated 105 patients with brain metastases, showing LDH >2ULN to be associated with poor outcomes. Namikawa et al. [56] analyzed 229 Japanese patients on ICI therapy, demonstrating significantly worse survival with elevated LDH. In a nationwide Danish cohort, Pedersen et al. [17] similarly found inferior OS for patients with high LDH. Rousset et al. [25], in 538 French patients treated with ICIs, observed shorter OS in patients with elevated LDH. In mucosal melanoma, Rozendorn et al. [41] reported a strong association between elevated LDH and reduced OS (HR 3.04, 95% CI 1.11–7.56, *p* < 0.05). Silasi et al. [42], in a Romanian real-world cohort of 127 patients, confirmed the adverse effect of high LDH. Sun et al. [19] studied 319 Chinese patients treated with PD-1 inhibitors, showing elevated LDH as a predictor of inferior OS. Finally, Zhou et al. [21] reported similar findings in 123 advanced melanoma patients on ICIs. The pooled random-effects analysis of MVA data confirmed elevated versus normal LDH as an adverse predictive factor for OS (HR 1.71, 95% CI 1.53–1.91, *p* < 0.001), with low heterogeneity (τ^2^ = 23.3%, *p* = 0.2080), with all pooled estimates consistently favoring worse survival in patients with elevated LDH (Figure 12).

Sensitivity analysis

Sensitivity analyses for elevated LDH and overall survival showed consistent results, with no single study materially affecting the pooled estimate. These findings are consistent with prior clinical and real-world evidence identifying LDH as an adverse prognostic marker in advanced melanoma.

UVA OS

**Figure 13:**
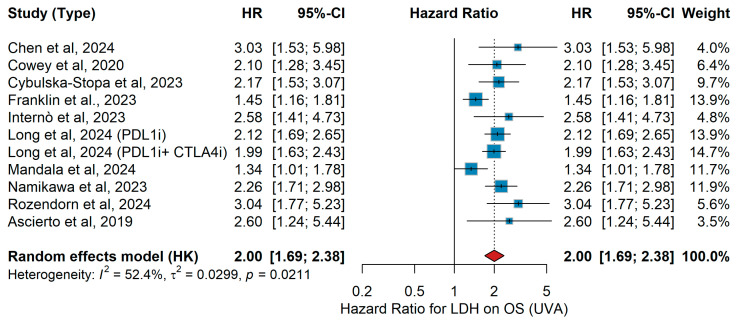
Forest plot of univariate analysis for overall survival (OS) in metastatic melanoma. LDH as a dichotomous variable. Elevated LDH levels were significantly associated with worse overall survival (OS).

In the univariate analysis (UVA), across the included studies, heterogeneous patient populations and treatment strategies were evaluated with respect to overall survival (OS). Cybulska-Stopa et al. [36] reported that elevated LDH was associated with worse OS. Franklin et al. [38] found a similar adverse effect. In the retrospective analysis by Internò et al. [39], high LDH remained a negative prognostic factor for OS. Long et al. [44] observed that elevated LDH predicted poorer OS both in the PD-L1 inhibitor group and in the PD-L1 + CTLA-4 inhibitor combination group. In the population studied by Mandala et al. [16], increased LDH correlated with inferior OS. Namikawa et al. [56] similarly demonstrated worse outcomes with elevated LDH. Rozendorn et al. [41] showed LDH to be an independent predictor of poor OS. In a Chinese cohort, Chen et al. [32] identified high LDH as an adverse prognostic marker (HR 3.03, 95% CI 1.53–5.98). Finally, Cowey [60] reported elevated LDH to be associated with significantly shorter OS. The pooled analysis of elevated versus normal LDH on OS, as depicted in the forest plot, yielded a combined HR 2.00 (95% CI 1.69–2.38; *p* = 0.0217; I^2^ = 52.4%, *p* = 0.0211, Figure 13).

MVA PFS

**Figure 14:**
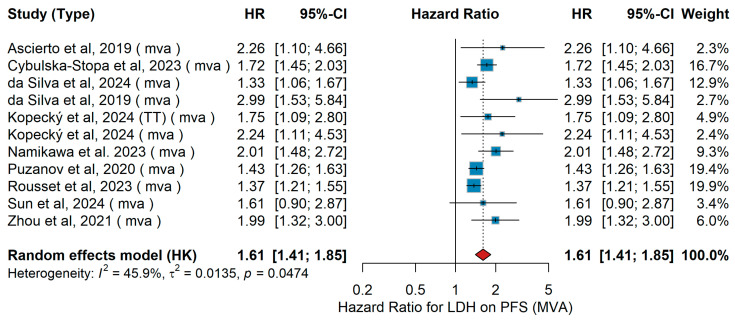
Forest plot of multivariate analysis for progression-free survival (PFS) in metastatic melanoma. LDH as a dichotomous variable. Elevated LDH levels were significantly associated with worse progression-free survival (PFS).

In the multivariate analysis (MVA), across the included studies, heterogeneous patient populations with advanced or metastatic melanoma were investigated, receiving either anti-PD-1 monotherapy (nivolumab, pembrolizumab), combinations with ipilimumab or sequential/targeted therapies. Ascierto et al. [23] examined 71 patients, largely pre-treated with ipilimumab, and reported significantly worse progression-free survival (PFS) for patients with elevated LDH. In the large real-world cohort by Cybulska-Stopa et al. [36] including 1037 first-line patients, elevated LDH was similarly associated with inferior PFS. Two analyses by da Silva [18,29] and colleagues showed consistent results: in a pooled international dataset of combination ipilimumab plus anti-PD-1, elevated LDH predicted shorter PFS, while in a detailed lesional-level analysis of 140 patients, LDH remained a strong adverse predictive factor (HR 2.99, 95% CI 1.53–5.84) [11]. Kopecký et al. [40] assessed both TT and immunotherapy cohorts, with LDH elevation associated with inferior outcomes in both groups. In a Japanese multicenter analysis of anti-PD-1-treated patients, Namikawa et al. [56] similarly found an adverse impact of LDH on PFS. In the pooled KEYNOTE trials analysis, Puzanov et al. [45] identified elevated LDH as a significant predictor of shorter PFS. The French multicenter study by Rousset et al. [25] (n > 600) also reported significantly worse PFS with elevated LDH. Sun et al. [19] observed a detrimental association in their prospective cohort, while Zhou et al. [21] in a Chinese cohort similarly confirmed elevated LDH as an independent adverse predictive factor. When pooled in a random-effects meta-analysis (Hartung–Knapp), elevated versus normal LDH was consistently associated with inferior PFS (HR 1.61, 95% CI 1.41–1.85, *p* = 0.047). Moderate heterogeneity was observed (τ^2^ = 0.0135, I^2^ = 45.9%), as shown in Figure 14.

Sensitivity Analysis

In multivariable models for LDH and progression-free survival, leave-one-out sensitivity analyses yielded HRs ranging from 1.57 to 1.67 for elevated versus normal LDH. No single study materially influenced the pooled estimate, indicating a stable association with poorer progression-free survival.

UVA PFS

**Figure 15:**
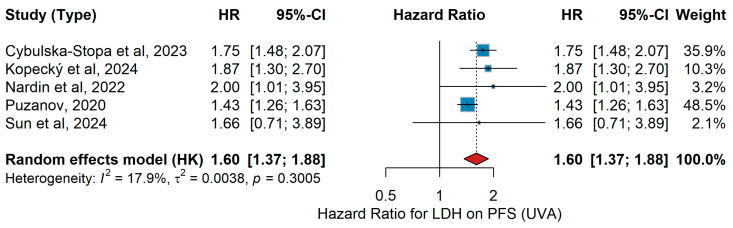
Forest plot of univariate analysis for progression-free survival (PFS) in metastatic melanoma. LDH as a dichotomous variable. Elevated LDH levels were significantly associated with worse progression-free survival (PFS).

In the univariate analysis (UVA), patients with advanced or metastatic melanoma harboring BRAF mutations were treated with either ΤΤ (BRAF/MEK inhibition) or immunotherapy (anti-PD-1 monotherapy or in combination with CTLA-4 blockade). In the large real-world cohort by Cybulska-Stopa et al. [36], first-line anti-PD-1 therapy with pembrolizumab or nivolumab (n = 1037) yielded a hazard ratio (HR) for progression-free survival (PFS) for elevated LDH levels. Similarly, Kopecký et al. [40] reported in 174 BRAF-mutant patients treated with either ΤΤ or anti-PD-1 that high LDH was associated with shorter PFS. Namikawa et al. [56], in a multicenter Japanese cohort of 336 patients, confirmed LDH as a negative prognostic factor. In a biomarker-oriented study, Nardin et al. [34] demonstrated that elevated LDH predicted inferior outcomes under immune checkpoint inhibitors. Puzanov et al. [45], pooling data from 1558 patients across three KEYNOTE trials, found that PFS is shorter in those with elevated LDH. Finally, in a Spanish retrospective series of 140 BRAF-mutant patients, Sun et al. [19] reported a similar trend. The pooled analysis using a random-effects model demonstrated that elevated LDH significantly predicted shorter PFS across studies, with HR 1.60 (95% CI 1.37–1.88, *p* < 0.001). Heterogeneity was low (I^2^ = 17.9%, *p* = 0.3005), as illustrated in Figure 15.

#### 3.2.4. Skin Disorders

Skin Disorder (Yes vs. No)
UVA OS

**Figure 16:**
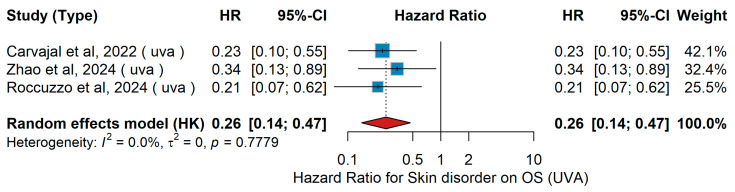
Forest plot of univariate analysis for overall survival (OS) in metastatic melanoma. Skin Disorder as a dichotomous variable. The presence of immune-related skin disorders was significantly associated with improved overall survival (OS).

In the univariable analysis (UVA), the predictive impact of skin disorder (no vs. yes) on overall survival was assessed. In the phase I study by Carvajal et al. [52], 42 patients with metastatic uveal melanoma were treated with tebentafusp. Zhao et al. [55], in a cohort of 55 patients with unresectable Stage IIIC–IV melanoma treated with PD-1 inhibitor plus IFN-α1b and anlotinib, reported a significant association for no versus yes. In the real-world study of Roccuzzo et al. [57] including 157 Stage III melanoma patients with positive sentinel lymph node biopsy, a significant association was also observed for no versus yes. The pooled random-effects model showed an HR of 0.26 (95% CI 0.14–0.47, *p* < 0.001) for the presence versus absence of skin disorder. Heterogeneity was negligible (τ^2^ = 0, Q = 0.78, *p* = 0.778, I^2^ = 0%), as shown in Figure 16.

UVA PFS

**Figure 17:**
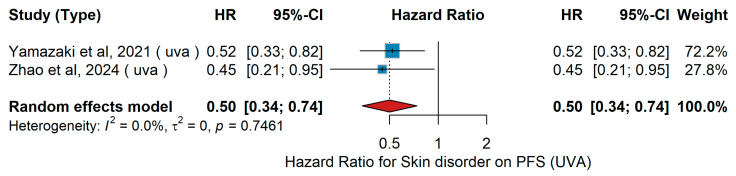
Forest plot of univariate analysis for progression-free survival (PFS) in metastatic melanoma. Skin disorder as a dichotomous variable. The presence of immune-related skin disorders was significantly associated with improved progression-free survival (PFS).

In the univariable analysis (UVA) for progression-free survival (PFS), Yamazaki et al. [47] reported in Japanese patients with advanced melanoma receiving nivolumab monotherapy that the occurrence of skin disorders as immune-related adverse events was associated with a significantly reduced risk of progression. Zhao et al. [55] examined Chinese patients with unresectable melanoma treated with second-line PD-1 antibody plus interferon-α1b and Anlotinib and found a protective effect of skin disorders. The pooled random-effects analysis confirmed a trend toward improved PFS in patients with skin disorders compared to those without (HR 0.50, 95% CI 0.34–0.74, *p* = 0.0005), with no evidence of heterogeneity (τ^2^ = 0.00; Q *p* = 0.7461; I^2^ = 0%), as shown in Figure 17.

#### 3.2.5. Ulceration

Ulceration (yes vs. no)
UVA OS

**Figure 18:**
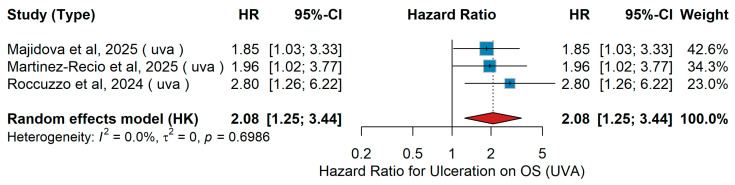
Forest plot of univariate analysis for overall survival (OS) in metastatic melanoma. Ulceration as a dichotomous variable. The presence of ulceration was significantly associated with worse overall survival (OS).

In the univariate analyses (UVA) of overall survival (OS), ulceration was consistently associated with worse prognosis across independent cohorts. Majidova et al. [27] studied 120 patients with Stage IIIB–IIID melanoma from 20 Turkish centers, of whom 56.7% had ulcerated primaries and received adjuvant therapy with nivolumab, dabrafenib plus trametinib, or interferon; ulceration was associated with inferior OS. Martinez-Recio et al. [70] evaluated 245 Spanish patients treated with anti-PD-1 adjuvant immunotherapy, with 56% presenting ulceration; OS was worse in the ulcerated subgroup. Similarly, in the Italian single-center study of 163 patients treated with adjuvant ΤΤ or anti-PD-1, Roccuzzo et al. [57] reported ulceration in 47.2% of cases, which was associated with a higher risk of death. When pooling these studies, ulceration versus non-ulceration was significantly associated with worse OS (pooled HR 2.08, 95% CI 1.25–3.44, *p* = 0.003). No heterogeneity was detected (τ^2^ = 0; Q = 0.72, *p* = 0.699; I^2^ = 0%). As illustrated in Figure 18, ulceration consistently conferred an adverse prognostic impact on overall survival.

UVA PFS

**Figure 19:**
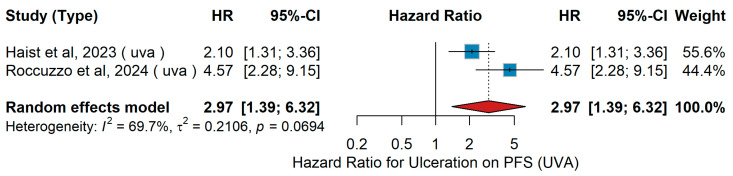
Forest plot of univariate analysis for progression-free survival (PFS) in metastatic melanoma. Ulceration as a dichotomous variable. The presence of ulceration was significantly associated with worse progression-free survival (PFS).

In the univariate analysis (UVA), the impact of ulceration (yes vs. no) on progression-free survival (PFS) was assessed across available cohorts. Haist et al. [48] investigated 515 patients with resected Stage III BRAF-mutant melanoma from the German ADOREG registry who had received adjuvant therapy with either anti-PD-1 agents or BRAF/MEK; in this population, ulceration was associated with an increased risk of progression. Similarly, Roccuzzo et al. [57] analyzed 163 patients with Stage III/IV-NED melanoma from an Italian single-center cohort who received adjuvant therapy with either dabrafenib + trametinib or anti-PD-1 agents; ulceration also emerged as a predictor of shorter PFS. When data from both studies were pooled in a random-effects meta-analysis, patients with ulceration tended to have worse progression-free survival (PFS) compared to those without ulceration (combined hazard ratio = 2.97, 95% CI 1.39–6.32, *p* = 0.005). Between-study heterogeneity was substantial (I^2^ = 69.7%, τ^2^ = 0.2106, Q test *p* = 0.0694). As illustrated in Figure 19, although effect sizes varied, the overall direction consistently favored worse PFS in ulcerated tumors.

#### 3.2.6. Tumor Mutational Burden (High vs. Low)

UVA PFS

**Figure 20:**
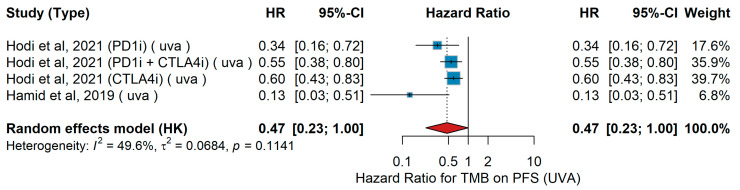
Forest plot of univariate analysis for progression-free survival (PFS) in metastatic melanoma. Tumor mutational burden as a dichotomous variable. High TMB was associated with improved progression-free survival (PFS).

In univariate analyses (UVAs) for progression-free survival (PFS), high versus low tumor mutational burden (TMB) was associated with differential outcomes across studies. In the phase Ia trial by Hamid et al. [59], including 45 patients with metastatic melanoma treated with atezolizumab (PD-L1 inhibitor), TMB-high patients had a significantly reduced risk of progression. In the phase III CheckMate 066 and 067 trials analyzed by Hodi et al. [49], patients received nivolumab monotherapy, nivolumab plus ipilimumab, or ipilimumab alone, and TMB-high status was consistently associated with longer PFS across treatment arms. When pooled across studies, TMB-high compared with TMB-low showed a trend toward improved PFS (pooled HR 0.47, 95% CI 0.33–0.7, *p* < 0.01). Moderate heterogeneity was observed (I^2^ = 49.6%, τ^2^ = 0.0684, *p* = 0.11), as shown in Figure 20.

Sensitivity analysis

Sensitivity analyses for tumor mutational burden and progression-free survival yielded HRs ranging from 0.37 to 0.55 across scenarios. The association remained consistently protective (HR < 1), with no single study materially affecting the pooled estimate.

### 3.3. Histologic/Pathologic Predictors

#### 3.3.1. Stage

Stage (III vs. IV)
MVA OS

**Figure 21:**
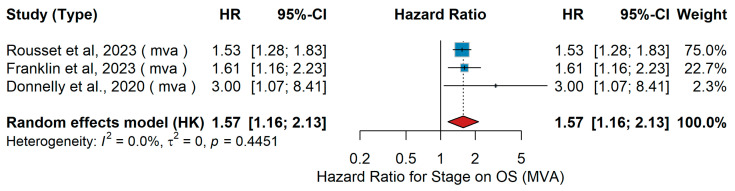
Forest plot of multivariate analysis for overall survival (OS) in metastatic melanoma. Stage as a dichotomous variable. Advanced stage was significantly associated with worse overall survival (OS).

In multivariate analyses (MVA) of Stage IV versus Stage III melanoma, three independent studies were included. Rousset et al. [25] examined 165 patients with metastatic melanoma of unknown primary site treated with PD-L1 inhibitor ± CTLA-4 inhibitor, reporting a significantly worse overall survival (OS) for Stage IV compared to Stage III disease. Franklin et al. [38] analyzed 1704 patients from the ADOREG registry, including both BRAF wild-type and BRAF V600 mutant populations treated with BRAF + MEK inhibitors or PD-1 inhibitor ± CTLA-4 inhibitor as first-line therapy, and similarly found inferior OS in Stage IV compared to Stage III. Donnelly et al. [74] evaluated 423 patients with metastatic melanoma treated with PD-L1 inhibitor ± CTLA-4 inhibitor and demonstrated an adverse impact of Stage IV disease relative to Stage III (HR 3.00, 95% CI 1.07–8.41, *p* = 0.04). The pooled analysis confirmed significantly worse OS in Stage IV compared to Stage III melanoma (HR 1.57, 95% CI 1.16–2.13, *p* < 0.05). Heterogeneity across studies was low (τ^2^ = 0; I^2^ = 0.0%; Q = 0.4451), as shown in Figure 21.

UVA OS

**Figure 22:**
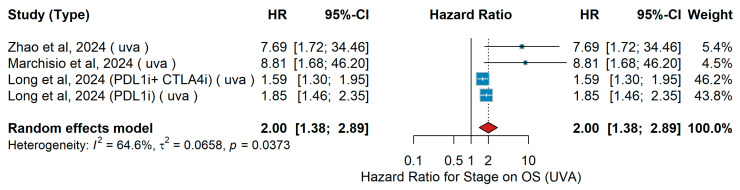
Forest plot of univariate analysis for overall survival (OS) in metastatic melanoma. Stage as a dichotomous variable. Advanced stage was significantly associated with worse overall survival (OS).

In the univariate analysis (UVA), several studies investigating melanoma populations across different stages and treatment contexts were included. Long et al. [44] analyzed immune checkpoint inhibitor (ICI)-naïve patients with unresectable or metastatic melanoma, comparing Stage IV versus Stage III disease in cohorts treated with nivolumab plus ipilimumab (NIVO + IPI) or nivolumab monotherapy, and reported worse overall survival (OS) for Stage IV patients. Marchisio et al. [28] examined patients with resected Stage III BRAF-mutated melanoma undergoing adjuvant targeted or immunotherapy and observed markedly worse OS in those exhibiting ctDNA positivity—representing higher stage-equivalent disease risk. Zhao et al. [55] evaluated Chinese patients with unresectable Stage IIIC–IV melanoma treated with PD-1 antibody plus IFN-α1b and anlotinib after prior immunotherapy failure, where advanced Stage IV disease was associated with poorer OS. The pooled random-effects analysis comparing Stage IV versus Stage III disease yielded an overall HR of 2.00 (95% CI 1.38–2.89, *p* <0.001), indicating a trend toward inferior survival for patients with advanced-stage melanoma. Between-study heterogeneity was moderate (I^2^ = 64.6%, τ^2^ = 0.0658, Q *p* = 0.037), as shown in Figure 22.

Sensitivity analysis

In univariate analyses for overall survival, disease stage showed substantial variability across leave-one-out analyses, with HRs ranging from 1.79 to 4.04. The exclusion of Long et al. [44] resulted in a marked increase in the pooled estimate, indicating sensitivity to individual large studies. While the direction of association remained consistent, the magnitude of the effect was unstable and should be interpreted with caution.

#### 3.3.2. Subtype

MVA OS

**Figure 23:**
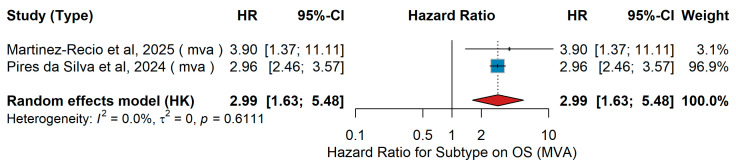
Forest plot of multivariate analysis for overall survival (OS) in metastatic melanoma. Subtype as a dichotomous variable. Unfavorable subtype was significantly associated with worse overall survival (OS), with no observed between-study heterogeneity.

In the multivariable analyses, Martinez-Recio et al. [70] evaluated 245 patients with resected Stage IIB–IV melanoma treated with adjuvant PD-1-based immunotherapy, showing significantly worse overall survival for acral compared with cutaneous melanoma (HR 3.90, 95% CI 1.37–11.11, *p* = 0.01). Similarly, Pires da Silva et al. [18] analyzed 533 patients with advanced melanoma and liver metastases treated with anti-PD-1 ± anti-CTLA-4 in the first-line setting and reported inferior survival for acral versus cutaneous subtypes (HR 2.96, 95% CI 2.46–3.57, *p* = 0.004). The pooled analysis from the forest plot confirmed a significantly worse prognosis for acral versus cutaneous melanoma, with a random-effects hazard ratio of 2.99 (95% CI 1.63–5.48, *p* < 0.001), as shown in Figure 23. Between-study heterogeneity was negligible (τ^2^ = 0.00, Q = 0.02, *p* = 0.61; I^2^ = 0.0%).

Subtype (acral vs. cutaneous)
UVA OS

**Figure 24:**
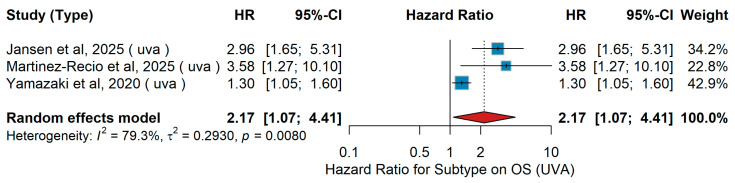
Forest plot of univariate analysis for overall survival (OS) in metastatic melanoma. Subtype as a dichotomous variable. Unfavorable subtype was significantly associated with worse overall survival (OS), with substantial between-study heterogeneity.

In the univariate analysis (UVA), three studies evaluated the association between melanoma subtype and overall survival (OS) in patients treated with PD-1-based immunotherapy across different clinical settings. Jansen et al. [14] included 374 patients with Stage IV melanoma who received first-line immune checkpoint inhibitors (anti-PD-1 monotherapy or anti-PD-1 plus anti-CTLA-4) and reported worse survival for acral compared with cutaneous melanoma. Martinez-Recio et al. [70] analyzed 245 patients with resected Stage IIB–IV melanoma treated with adjuvant PD-1-based immunotherapy, also showing inferior outcomes for acral compared with cutaneous primaries (HR 3.58, 95% CI: 1.27–10.10). In the Japanese CREATIVE study, Yamazaki et al. [46] assessed 124 patients with unresectable Stage III–IV melanoma treated with nivolumab and found a smaller but significant disadvantage for acral cases. The pooled random-effects analysis demonstrated a significantly increased risk of death for acral versus cutaneous melanoma (HR 2.17, 95% CI 1.07–4.41, *p* = 0.032), as shown in Figure 24. Heterogeneity across studies was considerable (I^2^ = 79.3%, τ^2^ = 0.2930, Q = 0.008). 

#### 3.3.3. Number of Metastatic Sites

Number of metastatic sites (>2 vs. ≤2)
MVA OS

**Figure 25:**
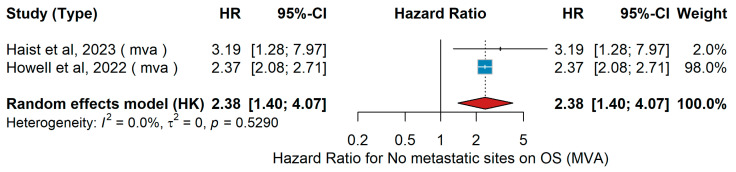
Forest plot of multivariate analysis for overall survival (OS) in metastatic melanoma. Number of metastatic sites as a dichotomous variable. A higher number of metastatic sites was significantly associated with worse overall survival, with no evidence of between-study heterogeneity.

Multivariable overall survival (MVA OS) analysis demonstrated that switching treatment modalities upon distant metastasis improved progression-free survival (median 9 vs. 5 months, *p* = 0.004) [48]. In a large SEER-Medicare cohort of older patients with metastatic or unresectable melanoma (n = 1435, mean age = 76.6 years, range ≥ 65), patients received CTLA-4 inhibitors PD-1 inhibitors, and 9.3% combination ICIs [53]. In this cohort, multivariable analysis showed that a greater number of metastatic sites was associated with increased mortality. In a separate multicenter retrospective study of adjuvant-treated patients who relapsed with distant metastases after PD-1 or ΤΤ (n = 237 out of 515; median age = 58 years; 56% men), 89% had cutaneous primaries, 48% ulcerated melanomas, and mean Breslow thickness = 3.6 mm [48]. Among these, multifocal disease with >2 metastatic sites was associated with markedly poorer survival (HR = 3.19, 95% CI 1.28–7.97, *p* = 0.013) in multivariate analysis. Pooled analysis in the present meta-analysis, comparing patients with >2 metastatic sites versus ≤2, demonstrated an overall HR = 2.38 (95% CI 1.4–4.07, *p* < 0.05), indicating significantly worse overall survival with increasing metastatic burden, as shown in Figure 25. No between-study heterogeneity was observed (τ^2^ = 0; I^2^ = 0%; Q *p* = 0.529).

UVA OS

**Figure 26:**
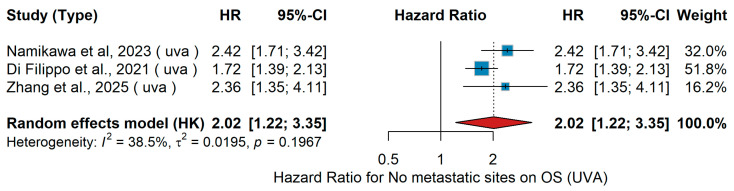
Forest plot of univariate analysis for overall survival (OS) in metastatic melanoma. Number of metastatic sites as a dichotomous variable. A higher number of metastatic sites was associated with significantly worse OS.

Univariable overall survival (UVA OS) analysis showed that a higher number of metastatic sites was significantly associated with worse overall survival across the included studies. In the study by Namikawa et al. [56], patients with >2 metastatic sites had poorer outcomes compared with those with ≤2 sites. Similarly, Di Filippo et al. [69] and Zhang et al. [20] reported inferior survival among patients with a higher metastatic burden. The pooled random-effects meta-analysis demonstrated a combined hazard ratio of 2.02 (95% CI 1.22–3.35, *p* < 0.05), indicating approximately a twofold increased risk of death in patients with >2 metastatic sites, as shown in Figure 26. Moderate heterogeneity was observed (I^2^ = 38.5%, τ^2^ = 0.0195, *p* = 0.1967).

#### 3.3.4. Brain Metastasis

Βrain metastasis (yes vs. no)
MVA OS

**Figure 27:**
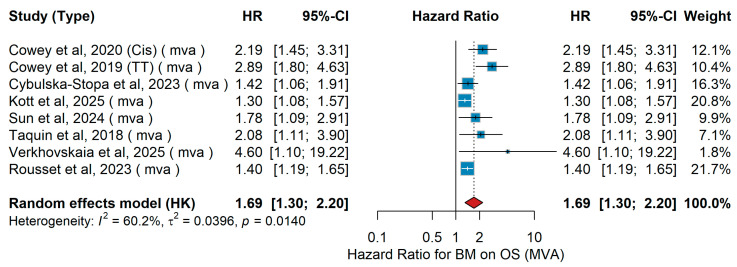
Forest plot of multivariate analysis for overall survival (OS) in metastatic melanoma. Brain metastasis as a dichotomous variable. The presence of brain metastases was associated with significantly worse OS.

A total of eight studies were included in the multivariable analysis investigating the association between brain metastases and overall survival (OS) in advanced melanoma. The pooled random-effects hazard ratio was 1.69 (95% CI 1.30–2.20, *p* = 0.014), indicating that the presence of brain metastases was significantly associated with worse overall survival, as shown in Figure 27. Between-study heterogeneity was moderate (I^2^ = 60.2%, τ^2^ = 0.0396, *p* = 0.014), suggesting variability in effect sizes likely related to differences in patient populations and treatment settings.

Sensitivity Analysis

In multivariable analyses comparing patients with versus without brain metastases, leave-one-out sensitivity analyses yielded HRs ranging from 1.53 to 1.84. The exclusion of Cowey et al. [61] slightly attenuated the pooled estimate, whereas the omission of Kott et al. [15] resulted in a modest increase, reflecting differences in individual study effect sizes. The exclusion of other studies had minimal impact, with the direction and magnitude of the association remaining stable.

MVA PFS

**Figure 28:**
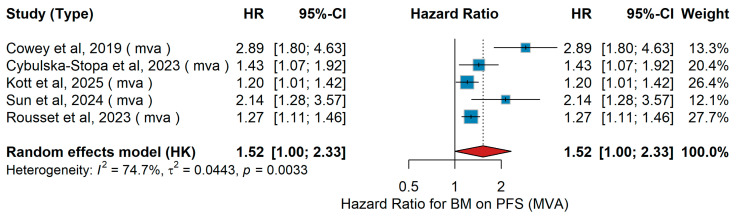
Forest plot of multivariate analysis for progression-free survival (PFS) in metastatic melanoma. Brain metastasis as a dichotomous variable. The presence of brain metastases was associated with significantly worse PFS.

A total of five studies were included in the multivariable analysis evaluating the impact of brain metastases on progression-free survival (PFS) in advanced melanoma. The pooled random-effects hazard ratio was 1.52 (95% CI 1.00–2.33, *p* = 0.05), suggesting a borderline significant association between the presence of brain metastases and worse PFS, as shown in Figure 28. Individual study estimates ranged from 1.20 to 2.89. Between-study heterogeneity was substantial (I^2^ = 74.7%, τ^2^ = 0.0443, *p* = 0.0033), indicating considerable variability across patient populations and treatment settings.

Sensitivity Analysis

In multivariable analyses of progression-free survival comparing patients with versus without brain metastases, leave-one-out sensitivity analyses yielded HRs ranging from 1.32 to 1.72. The association consistently indicated a higher risk of progression for patients with brain metastases.

UVA OS

**Figure 29:**
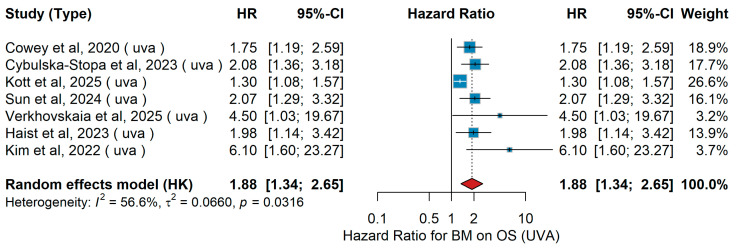
Forest plot of univariate analysis for overall survival (OS) in metastatic melanoma. Brain metastasis as a dichotomous variable. The presence of brain metastases was associated with significantly worse OS.

A total of seven studies were included in the univariate analysis evaluating the association between brain metastases and overall survival (OS). The pooled random-effects hazard ratio was 1.88 (95% CI 1.34–2.65, *p* = 0.0316), indicating that the presence of brain metastases was significantly associated with worse overall survival, as shown in Figure 29. Individual study estimates consistently supported a negative prognostic role, although effect sizes varied across studies. Between-study heterogeneity was moderate (I^2^ = 56.6%, τ^2^ = 0.0660, *p* = 0.0316), likely reflecting differences in treatment regimens and patient characteristics.

Sensitivity Analysis

In univariate analyses comparing patients with versus without brain metastases, leave-one-out sensitivity analyses yielded HRs ranging from 1.82 to 2.05. The association consistently indicated poorer overall survival for patients with brain metastases.

UVA PFS

**Figure 30:**
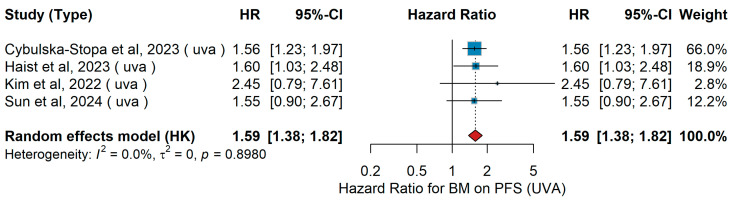
Forest plot of univariate analysis for progression-free survival (PFS) in metastatic melanoma. Brain metastasis as a dichotomous variable. The presence of brain metastases was associated with significantly worse PFS.

A total of four studies were included in the univariate analysis evaluating the association between brain metastases and progression-free survival (PFS). The pooled random-effects hazard ratio was 1.59 (95% CI 1.38–1.82, *p* = 0.001), indicating that the presence of brain metastases was significantly associated with worse PFS, as shown in Figure 30. Between-study heterogeneity was negligible (I^2^ = 0.0%, τ^2^ = 0, *p* = 0.898), demonstrating highly consistent effect estimates across studies.

Sensitivity Analysis

In univariate analyses, HRs ranged from 1.57 to 1.64 across leave-one-out sensitivity analyses, with minimal variation. The association consistently indicated poorer progression-free survival for patients with brain metastases.

#### 3.3.5. Liver Metastasis

Liver Metastasis (yes vs. no)
MVA OS

**Figure 31:**
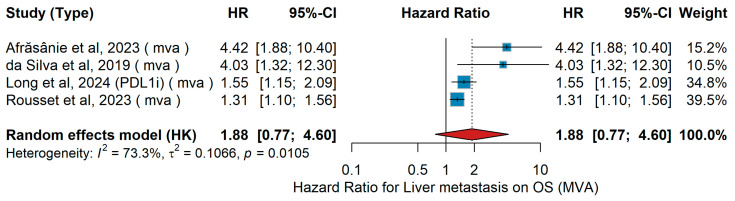
Forest plot of multivariate analysis for overall survival (OS) in metastatic melanoma. Liver metastasis as a dichotomous variable. The presence of liver metastases was associated with worse OS, although the pooled effect did not reach statistical significance.

Meta-analysis of multivariable-adjusted overall survival (MVA OS) was conducted according to the presence vs. absence of liver metastases. In the Romanian real-world cohort by Afrăsânie et al. [37], 51 treatment-naïve metastatic melanoma patients received first-line nivolumab, with liver metastases identified as independent predictive factors for poor survival. In contrast, the Australian multicenter analysis by da Silva et al. [29] included 140 patients treated with first-line ipilimumab plus anti-PD-1 (nivolumab or pembrolizumab); patients with liver metastases had significantly inferior OS. The pooled analysis by Long et al. [44], comprising 1375 ICI-naïve advanced melanoma patients across six CheckMate trials, demonstrated prolonged OS with nivolumab plus ipilimumab versus nivolumab alone, while the presence of liver metastases under nivolumab monotherapy was associated with worse outcomes. Finally, in the French MELBASE prospective cohort, Rousset et al. [25] compared 265 patients with melanoma of unknown primary site (MUP) to 1617 with a cutaneous primary site and found that patients with liver metastases had poorer OS. In the present meta-analysis, the pooled random-effects hazard ratio for the impact of liver metastases on overall survival was 1.88 (95% CI 0.77–4.60), indicating a trend toward worse survival in patients with liver metastases; however, this association did not reach statistical significance, as shown in Figure 31. Between-study heterogeneity was substantial (I^2^ = 73.3%, τ^2^ = 0.1066, *p* = 0.0105), reflecting considerable variability in effect estimates across studies.

UVA OS

**Figure 32:**
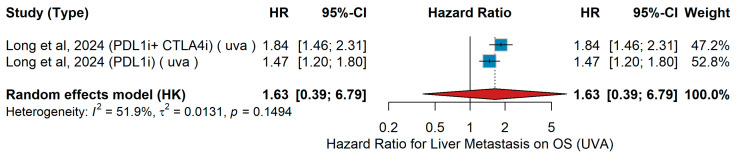
Forest plot of univariate analysis for overall survival (OS) in metastatic melanoma. Liver metastasis as a dichotomous variable. The presence of liver metastases was associated with worse OS, although the pooled effect was not statistically significant.

Meta-analysis of univariable overall survival (UVA OS) was conducted according to the presence versus absence of liver metastases. The pooled analysis from Long et al. [44] evaluated the prognostic impact of liver metastases in advanced melanoma patients treated with immune checkpoint inhibitors (ICIs). In the univariable analysis, patients receiving combined PD-L1 and CTLA-4 blockade (PD-L1i + CTLA4i) had significantly worse overall survival when liver metastases were present. Similarly, in the cohort treated with PD-L1 inhibitor monotherapy (PD-L1i), liver involvement remained an adverse prognostic factor. When pooled using a random-effects model, the combined hazard ratio for the impact of liver metastases on OS was 1.63 (95% CI: 0.39–6.79), indicating a trend toward poorer outcomes, though this association was not statistically significant, as shown in Figure 32. Moderate heterogeneity was observed across studies (I^2^ = 51.9%, τ^2^ = 0.0131, *p* = 0.1494).

Sensitivity analyses

Sensitivity analyses excluding CTLA-4 monotherapy cohorts confirmed that the pooled estimates remained robust.

UVA PFS

**Figure 33:**
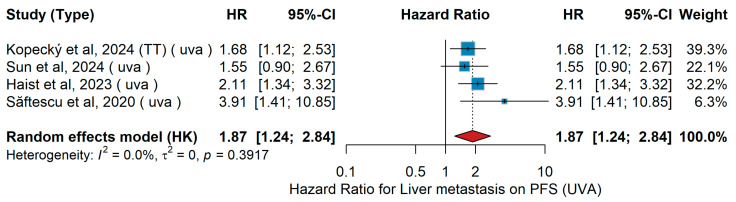
Forest plot of univariate analysis for progression-free survival (PFS) in metastatic melanoma. Liver metastasis as a dichotomous variable. The presence of liver metastases was associated with significantly worse PFS.

In the univariable analysis (UVA) for progression-free survival (PFS), the impact of liver metastasis (yes vs. no) was evaluated across four studies. Kopecký et al. [40], (TT population) reported that the presence of liver metastases was associated with shorter PFS. Similarly, Sun et al. [19] analyzed a different patient cohort and observed a trend toward worse PFS among patients with liver involvement. Haist et al. [48] also found that liver metastases were linked to poorer outcomes, while Săftescu et al. [35] observed a particularly strong association between liver metastases and inferior PFS. The pooled analysis using a random-effects model (Hartung–Knapp adjustment) demonstrated a combined hazard ratio of 1.87 (95% CI: 1.24–2.84), as shown in Figure 33. Heterogeneity across studies was negligible (I^2^ = 0.0%, τ^2^ = 0, Q-test *p* = 0.3917).

Importantly, these findings are consistent in direction and magnitude with both the multivariable (MVA) and univariable (UVA) meta-analyses of overall survival (OS), which similarly identified liver metastases as an adverse prognostic factor. Across independent cohorts—including real-world and clinical trial populations—liver involvement was repeatedly associated with poorer survival outcomes under immune checkpoint inhibitor (ICI) therapy [11,37,44,77]. This concordance between PFS and OS analyses reinforces the robustness of the observed negative prognostic impact of liver metastases in advanced melanoma.

### 3.4. Molecular Predictors

#### 3.4.1. PD-L1

PD-L1 Status (high vs. low)
MVA PFS

**Figure 34:**
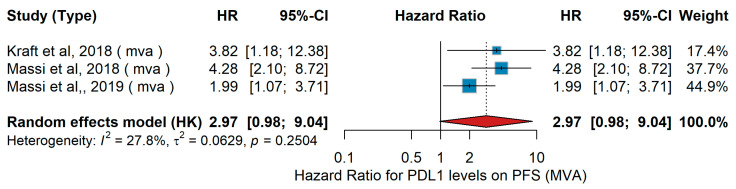
Forest plot of multivariate analysis for progression-free survival (PFS) in metastatic melanoma. PD-L1 Status as a dichotomous variable. Higher PD-L1 expression was not significantly associated with worse PFS.

A total of three studies were included in the multivariate analysis (MVA) investigating the association between PD-L1 expression levels and progression-free survival (PFS). Across the three studies, a total of 210 melanoma patients were analyzed. In the study by Kraft et al. [63], 66 patients with desmoplastic melanoma were included, with a median age of 70 years and a male-to-female ratio of approximately 7:4. Most tumors were located on the head and neck, and none of the patients had received prior systemic therapy. In Massi et al. [66] the cohort consisted of 80 metastatic melanoma patients harboring BRAF V600 mutations, all treated with BRAF inhibitors—vemurafenib (n = 46) or dabrafenib (n = 34). The median age was 56 years (range 21–86), with 53% males, and 56% of patients presented with Stage M1C disease. In Massi et al. [65], 64 BRAF-mutated metastatic melanoma patients were evaluated; 39 received BRAF inhibitor monotherapy, while 25 were treated with a combination of BRAF and MEK inhibitors. The median age was again 56 years, with 56% males and 53% at Stage M1C. Overall, these studies included predominantly male patients with advanced or desmoplastic melanoma, with median ages between 56 and 70 years, and treatment groups mainly based on BRAF-targeted therapy regimens. The pooled hazard ratio (HR) for high versus low PD-L1 expression was 2.97 (95% CI: 0.98–9.04). The pooled hazard ratio (HR) for high versus low PD-L1 expression was 2.97 (95% CI: 0.98–9.04), as shown in Figure 34. The pooled analysis shows a trend toward poorer PFS in patients with high PD-L1 expression compared to those with low expression, which did not reach statistical significance. Between-study heterogeneity was low (I^2^ = 27.8%, τ^2^ = 0.0629, Q = 2.55, *p* = 0.25).

#### 3.4.2. Circulating Tumor DNA (High Versus Low)

MVA OS

**Figure 35:**
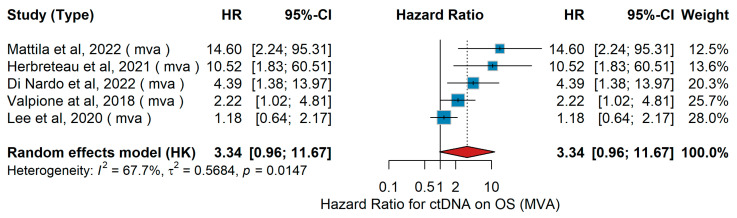
Forest plot of multivariate analysis for overall survival (OS) in metastatic melanoma. Circulating tumor DNA as a dichotomous variable. The presence of detectable ctDNA was not significantly associated with worse OS.

In this multivariable analysis (MVA), several studies assessed the predictive impact of circulating tumor DNA (ctDNA) on overall survival (OS) in advanced melanoma. Across five studies (≈340 patients), all participants had advanced or metastatic disease treated with modern systemic therapies, including immune checkpoint inhibitors and ΤΤ. Herbreteau et al. [77] analyzed 102 patients with Stage III–IV BRAF- or NRAS-mutated melanoma receiving anti-PD-1 therapy, showing that baseline ctDNA positivity at treatment initiation predicted poorer OS. Mattila et al. [51] examined 19 metastatic cases in the COBRA trial and found that high pre-treatment ctDNA levels and detectability at best response were associated with inferior OS. Di Nardo et al. [43] evaluated 48 patients treated with BRAF/MEK inhibitors or immunotherapy, reporting shorter OS with higher baseline ctDNA. In patients with brain metastases, Lee et al. [71] did not find a significant association between ctDNA and OS, while Valpione et al. [64] demonstrated that elevated cfDNA levels correlated with poorer survival. The pooled forest analysis comparing high versus low ctDNA confirmed the adverse predictive impact on OS (HR = 3.34, 95% CI 0.96–11.67, *p* = 0.06), as shown in Figure 35, with significant heterogeneity (I^2^ = 67.7%, τ^2^ = 0.5684, *p* = 0.0147).

UVA OS

**Figure 36:**
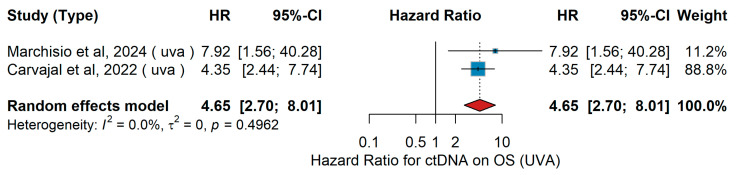
Forest plot of univariate analysis for overall survival (OS) in metastatic melanoma. Circulating tumor DNA as a dichotomous variable. The presence of detectable ctDNA was associated with significantly worse OS.

In the univariable analysis (UVA), only two studies were available, which limits the robustness of the pooled estimate and increases the uncertainty of the meta-analysis. Despite this limitation, the direction of effect was consistent with the multivariable analysis (MVA), supporting the adverse prognostic impact of detectable ctDNA on overall survival (OS). Marchisio et al. [28] analyzed a cohort of patients with uveal melanoma treated with systemic therapy, reporting that detectable ctDNA was associated with poorer OS. Similarly, Carvajal et al. [52] examined a comparable population receiving systemic treatment and observed inferior survival among ctDNA-positive patients. The pooled analysis using a random-effects model yielded an HR of 4.65 (95% CI 2.70–8.01, *p* < 0.01), as shown in Figure 36, indicating a significant adverse effect of ctDNA positivity on overall survival. Heterogeneity across studies was low (I^2^ = 0.0%, τ^2^ = 0, *p* = 0.4962).

MVA PFS

**Figure 37:**
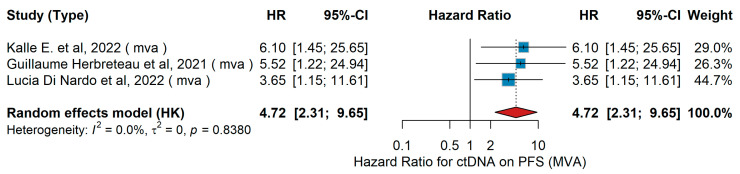
Forest plot of multivariate analysis for progression-free survival (PFS) in metastatic melanoma. Circulating tumor DNA as a dichotomous variable. Detectable ctDNA was associated with significantly worse PFS.

In the multivariable analysis (MVA) for progression-free survival (PFS), the prognostic impact of detectable circulating tumor DNA (ctDNA) was evaluated across three studies. Kalle E. Mattila et al. [51], 2020 reported that the presence of ctDNA was significantly associated with shorter PFS. Similarly, Herbreteau et al. [77] found that detectable ctDNA predicted poorer PFS, while Di Nardo et al. [43] observed a comparable association. The pooled analysis using a random-effects model demonstrated a combined hazard ratio of 4.72 (95% CI: 2.31–9.65, *p* < 0.001), as shown in Figure 37, confirming the adverse prognostic value of ctDNA detectability for PFS. Heterogeneity among studies was negligible (I^2^ = 0.0%, τ^2^ = 0, Q-test *p* = 0.8380).

Sensitivity Analysis

In multivariable analyses of ctDNA and overall survival, leave-one-out sensitivity analyses yielded HRs ranging from 2.59 to 4.64. While all estimates indicated poorer overall survival with detectable ctDNA, the magnitude of the effect varied substantially, indicating sensitivity to individual studies.

## 4. Discussion

Over the past decade, immunotherapy has profoundly transformed the therapeutic landscape of metastatic melanoma, establishing immune checkpoint inhibitors (ICIs) as the cornerstone of modern management and achieving unprecedented improvements in survival. Nonetheless, clinical benefit remains highly variable, with a significant proportion of patients experiencing primary or acquired resistance. This variability underscores the pressing need to delineate predictive factors that can guide treatment selection and optimize outcomes. Emerging evidence suggests that a spectrum of clinicopathological features, including patient age, performance status, and serum biomarkers, alongside histological patterns and molecular signatures, may influence response to immunotherapy. With the continuous expansion of the therapeutic landscape through innovative agents and neoadjuvant strategies, establishing robust predictive markers is becoming indispensable for optimizing patient stratification and advancing personalized treatment in melanoma.

Building on this background, in this systematic review and meta-analysis, we synthesized evidence from 63 studies encompassing 73,140 patients with metastatic melanoma, evaluating clinical, laboratory, molecular, and histopathological characteristics in relation to treatment outcomes. By systematically integrating these factors, we aimed to clarify their potential predictive role and to assess their impact on overall survival (OS) and progression-free survival (PFS) in patients receiving immunotherapy. This comprehensive approach provides a framework to better understand how baseline patient- and tumor-related features may shape therapeutic response and long-term prognosis.

Regarding demographic characteristics, gender did not emerge as a reliable predictor of immunotherapy efficacy in advanced melanoma. Although seven studies reported statistically significant associations, their directions of effect were inconsistent, and the between-study heterogeneity was extreme. Even when restricting to multivariable analyses, hazard ratios ranged from a strikingly elevated risk in females within a very small cohort to a modest protective effect in larger populations. Comparable contradictions were observed for progression-free survival, where significant but opposing results precluded meaningful quantitative synthesis. Our findings are consistent with the existing literature showing no robust evidence that sex modifies the efficacy of immunotherapy in metastatic melanoma. Large registry-based analyses, such as the Dutch Melanoma Treatment Registry [68], demonstrated comparable safety and survival outcomes between men and women after immune checkpoint inhibition, despite baseline clinical differences. Similarly, a U.S. national cohort study [78] reported no sex-related differences in receipt or survival benefit from immunotherapy. Finally, a systematic review and meta-analysis of randomized trials across cancers [79] confirmed overall survival benefit in both sexes, with melanoma specifically showing no consistent gender effect. Taken together, these data reinforce our observation that sex does not represent a reliable predictive factor for immunotherapy response in melanoma.

In contrast to gender, our analysis shows that older age is associated with poorer overall survival in patients with metastatic melanoma treated with immunotherapy. While age analyzed as a continuous variable did not yield a significant effect, the categorical analysis consistently indicated worse outcomes among older patients, with moderate heterogeneity across studies supporting the robustness of this association. Sensitivity analysis confirmed the stability of these results, showing consistent hazard ratios across studies and no substantial heterogeneity. Our findings are consistent with the meta-analysis by Li et al. [80], which also found that younger patients experience greater benefit from immunotherapy-based strategies. Taken together, the evidence suggests that advancing age may limit the efficacy of immunotherapy, likely due to a combination of immunosenescence, increased susceptibility to treatment-related toxicities, and reduced physiological reserve, factors that collectively compromise both treatment tolerance and long-term survival in elderly patients [81,82]. Therefore, further investigation is warranted to better characterize treatment tolerance in this population and to guide the development of tailored therapeutic approaches for older patients.

Consistent with the effect of age, ECOG performance status consistently emerged as one of the most robust prognostic factors for outcomes in metastatic melanoma treated with immunotherapy. Whether comparing patients with optimal functional status to those with even mild impairment, or those with moderate to severe impairment to better-preserved counterparts, the direction of association was uniform: poorer functional status was linked to reduced clinical benefit. This pattern was evident for both overall survival and progression-free survival, and it was maintained across analyses that accounted for confounding factors as well as in unadjusted comparisons. Although some variability was observed in the magnitude of the effect and heterogeneity was considerable in certain settings, the consistency of the trend underscores the clinical relevance of performance status. Furthermore, sensitivity analyses supported the consistency and reliability of these findings across different study designs, reinforcing performance status as a dependable prognostic indicator in this context. In line with our findings, a recent large-scale meta-analysis of randomized trials by Li and colleagues demonstrated that patients with an ECOG of 0 experienced significantly greater benefit from immunotherapy, particularly in combination regimens, whereas those with impaired performance status derived markedly less survival advantage, further reinforcing ECOG as a critical determinant of immunotherapy outcomes in advanced melanoma [80]. Furthermore, meta-analyses across multiple solid tumors (beyond melanoma) show that immunotherapy, whether as monotherapy or in combination, provides survival benefits in both ECOG 0 and 1 populations [83]. Taken together, these findings highlight ECOG performance status as a critical determinant of immunotherapy outcomes in metastatic melanoma, reinforcing the importance of comprehensive clinical assessment prior to treatment initiation and supporting its incorporation into future prognostic algorithms, while also underscoring the need to further evaluate its role in more frail patient groups.

Shifting the focus from functional capacity to somatic profile, emerging evidence suggests that body composition could influence patient outcomes under immunotherapy. Our meta-analysis indicates that a higher BMI prior to the initiation of immunotherapy may confer a survival advantage in patients with advanced melanoma. Although the effect was attenuated when restricted to multivariable models, the pooled evidence from both univariable and multivariable analyses suggests that overweight or obese patients exhibit improved overall survival compared with their normal-weight counterparts. The consistency of findings across independent cohorts and the very low degree of heterogeneity strengthen the robustness of this observation. Moreover, sensitivity analyses confirmed the robustness of these findings, further supporting the reliability of the observed association. This survival benefit can be interpreted within the framework of the “obesity paradox,” whereby excess adiposity, despite its established protumorigenic effects, appears to enhance the efficacy of immune checkpoint blockade. Obesity-related immunometabolic alterations such as chronic low-grade inflammation and increased adipokine secretion might reshape the tumor microenvironment and could drive T-cell exhaustion, a state that would become particularly responsive to PD-1/PD-L1 inhibition. Supporting evidence includes higher tumor-infiltrating lymphocytes and lower circulating immune checkpoints in obese patients, as well as preclinical data showing that leptin-induced T-cell dysfunction can be reversed by checkpoint blockade [53,84]. Collectively, these mechanisms provide a biologically plausible rationale for the improved overall survival observed in overweight and obese melanoma patients treated with immunotherapy. However, given the attenuation of significance in multivariable analyses and the observational nature of the included studies, residual confounding and selection bias cannot be excluded. Also, we were not able to detect data to exclude cases of sarcopenic obesity, where BMI could be high, but muscle mass would be low, which is a known adverse prognostic factor. Thus, while BMI emerges as a promising and easily measurable prognostic factor, further mechanistic studies and prospective validation are required before it can be integrated into clinical decision-making.

Transitioning from patient-related to circulating biomarkers, serum indicators of tumor metabolism have also demonstrated prognostic relevance. In this regard, elevated baseline LDH emerged as a consistently adverse prognostic factor in advanced melanoma treated with immune checkpoint inhibitors, being associated with inferior overall and progression-free survival across both univariate and multivariate analyses. The robustness of this finding was supported by its consistency across diverse cohorts and treatment modalities, with only limited heterogeneity observed, suggesting that variability in effect size primarily reflects differences in study populations rather than inconsistency of the marker itself. Moreover, sensitivity analyses demonstrated that the associations between elevated LDH and both progression-free and overall survival remained consistent across leave-one-out models, indicating that the observed effects were not driven by any single study. These results are in line with prior meta-analyses, including those by Patrelli Fausto et al. and Xu Jun et al., which similarly demonstrated that elevated LDH predicts poorer outcomes in melanoma patients receiving immunotherapy or targeted therapies [85,86]. A possible explanation for this association is the link between high LDH levels and a glycolysis-dominant tumor phenotype driven by the Warburg effect, whereby melanoma cells preferentially rely on aerobic glycolysis for energy production. By converting pyruvate to lactate, LDH sustains this metabolic shift but also increases lactate accumulation, which acidifies the tumor microenvironment [87,88]. This acidic milieu could suppress key antitumor immune responses by impairing the function of CD8^+^ T cells and natural killer cells, while favoring the activity of immunosuppressive cell populations [89]. Experimental studies further support this mechanism, showing that LDH expression is elevated in melanoma and that its inhibition reduces lactate production, cell proliferation, and tumor growth [87]. Collectively, these findings suggest that LDH not only reflects aggressive tumor biology but may also contribute to an immunosuppressive microenvironment, thereby providing a coherent explanation for its strong prognostic impact in patients undergoing immunotherapy.

Expanding the focus from biochemical to disease burden markers, our findings indicate a consistent association between metastatic burden and overall survival in patients with metastatic melanoma treated with immune checkpoint inhibitors. Patients with a higher number of metastatic sites prior to immunotherapy initiation tended to have less favorable survival outcomes compared with those with limited disease spread, suggesting that metastatic extent serves as a prognostic indicator reflecting tumor aggressiveness and disease biology. Patients with a high metastatic burden may exhibit reduced responsiveness to immune checkpoint inhibitors due to the cumulative immunosuppressive effects arising from multiple metastatic microenvironments. The coexistence of several metastatic sites generates spatial heterogeneity in immune regulation, promoting immune escape through expansion of regulatory T cells, dysfunction of cytotoxic CD8^+^ T lymphocytes, impaired antigen presentation, and dysregulated cytokine signaling. These mechanisms collectively dampen systemic antitumor immunity and T-cell-mediated cytotoxicity. Moreover, increased metastatic load often reflects greater tumor mass and advanced immune exhaustion, accompanied by activation of inhibitory signaling pathways and exclusion of effector T cells from the tumor niche [90,91]. Consequently, patients with widespread disease display a globally suppressed immune state that limits the efficacy of PD-1/PD-L1 and CTLA-4 blockade. Further prospective studies are warranted to determine whether metastatic burden functions solely as a prognostic marker or also modulates intrinsic responsiveness to immunotherapy.

In relation to specific metastatic sites, the presence of liver metastases was not significantly associated with inferior outcomes among patients with advanced melanoma treated with immune checkpoint inhibitors. Although a modest trend toward reduced overall and progression-free survival was observed, this did not reach statistical significance, suggesting that hepatic involvement does not preclude clinical benefit from immunotherapy. Sensitivity analyses revealed moderate variability across studies, with large pooled cohorts exerting the greatest influence on effect estimates. While the direction of association remained consistent, the magnitude of risk appeared context-dependent, reflecting differences in study design and patient composition. From a biological perspective, these findings may seem counterintuitive given that liver metastases are known to foster an immunosuppressive microenvironment. The liver’s inherent tolerogenic milieu—shaped by constant exposure to gut-derived antigens and secretion of cytokines such as IL-10 and TGF-β—promotes regulatory T-cell activation and attenuates cytotoxic T-cell function [92,93]. Nonetheless, our analysis did not confirm a significant clinical impact of hepatic involvement on immunotherapy efficacy. Variability in patient characteristics, treatment regimens, and methodological design across studies, together with evolving therapeutic strategies and differences in melanoma subtypes, may partly explain these discrepancies. Overall, these results suggest that while hepatic metastases may exert local immunosuppressive effects, they do not independently predict lack of benefit from immune checkpoint inhibition in current clinical practice.

By contrast, brain metastases in advanced melanoma are consistently associated with inferior outcomes under immune checkpoint inhibition, with both overall and progression-free survival significantly compromised. While the adverse effect was uniform, heterogeneity across studies likely reflects differences in patient selection, prior therapies, and treatment strategies. Sensitivity analyses further substantiated these findings, showing only minor variation in effect estimates across leave-one-out iterations. The exclusion of individual large cohorts modestly shifted the pooled estimates but did not alter the overall direction or significance of the association. These results align with the biological complexity of intracranial disease, where tumor–immune interactions within the central nervous system and the restricted penetrance of systemic agents across the blood–brain barrier reduce therapeutic efficacy [94]. Supporting this, the meta-analysis of Evan et al. showed that although immunotherapy and targeted agents have improved survival in melanoma brain metastases, median survival remains considerably shorter than in patients without intracranial involvement [95]. Similarly, Long et al. [96] reported that combination immunotherapy achieves durable intracranial responses mainly in asymptomatic patients, whereas outcomes are substantially poorer in those with neurological symptoms or corticosteroid use [96]. A likely explanation for these findings lies in the unique tumor microenvironment of brain metastases: unlike other malignancies with CNS-penetrant targeted options, such as EGFR- or ALK-driven lung cancer, systemic immunotherapies remain less efficacious in melanoma [97,98]. Moreover, the brain metastatic niche is characterized by immunosuppressive cellular components that downregulate key co-stimulatory molecules (e.g., CD80, CD86, CD40), impair antigen presentation, and disrupt local homeostasis. In this highly suppressive landscape, tumor-infiltrating lymphocytes are scarce and functionally impaired compared to primary melanoma, further diminishing the effectiveness of immune checkpoint inhibition [99]. Collectively, these insights emphasize that immunotherapy is most effective in asymptomatic brain metastases when immune function is preserved, while the immunosuppressive brain microenvironment and systemic barriers significantly constrain therapeutic benefit, highlighting the need for innovative strategies to overcome these challenges.

Immune-related manifestations during treatment can also carry important prognostic information. The presence of immune-related skin disorders during immunotherapy appears to be a favorable prognostic indicator in patients with metastatic melanoma. Our meta-analysis demonstrated that patients who developed dermatologic immune-related adverse events exhibited improved overall survival and a trend toward longer progression-free survival compared with those who did not experience such manifestations. Notably, the absence of heterogeneity across studies reinforces the robustness and consistency of this association, suggesting that the prognostic value of cutaneous immune manifestations remains stable across diverse study populations and therapeutic contexts. The consistent link between cutaneous immune-related adverse events (irAEs) and favorable outcomes in patients receiving immune checkpoint inhibitors (ICIs) likely reflects a shared immunologic basis. Histopathologic analyses have shown that eruptions such as maculopapular rashes contain dense perivascular CD4^+^ and CD8^+^ T-cell infiltrates, indicative of strong T-cell-mediated activation [100]. Similarly, psoriasiform and lichenoid reactions are associated with a Th1/Th17-polarized cytokine milieu enriched in TNF-α, IL-2, IL-6, IL-17, and IFN-γ—cytokines that also underpin effective antitumor immune responses [101,102]. Moreover, ICI-induced vitiligo exemplifies antigenic cross-reactivity, whereby cytotoxic T cells recognize shared melanocytic and melanoma antigens such as tyrosinase, MART-1, and GP100 [103]. Collectively, these mechanistic insights suggest that cutaneous irAEs serve as visible markers of systemic immune activation, in which self-directed T-cell responses mirror enhanced tumor recognition and elimination. This biological coherence may explain the consistent prognostic advantage observed in our analysis and highlights the potential of skin immune manifestations as accessible biomarkers of therapeutic efficacy in melanoma immunotherapy.

From a pathological standpoint, the presence of ulceration was consistently associated with inferior overall survival in patients treated with immune checkpoint inhibitors. The absence of heterogeneity across studies strengthens the robustness of this association, suggesting that ulceration exerts a uniform adverse impact on long-term outcomes irrespective of cohort characteristics or treatment setting. These clinical findings may be biologically explained by the distinct immunosuppressive and molecular landscape observed in ulcerated melanomas. Patients with ulcerated melanoma may exhibit a reduced response to immune checkpoint blockade due to a constellation of immunoregulatory alterations within the tumor microenvironment. Ulceration has been associated with increased infiltration of regulatory T cells and M2-polarized macrophages, together with a relative depletion of cytotoxic CD8^+^ and GZMB^+^ T cells, thereby fostering an immunosuppressive milieu [104]. At the molecular level, ulcerated, immune-depleted tumors demonstrate downregulation of HLA class I and II genes and reduced expression of immune checkpoint molecules, leading to impaired antigen presentation and suboptimal T-cell activation [105]. Moreover, recurrent copy-number losses affecting immune-regulatory genes and overexpression of EIF3B—linked to attenuated T-cell receptor signaling, lower immune infiltration, and enhanced TGF-β1 secretion—further reinforce mechanisms of immune evasion [106,107]. Collectively, these findings support the notion that ulceration reflects an immunologically “cold” tumor phenotype characterized by diminished antitumor immune activity, which may account for the reduced efficacy of immunotherapy and poorer clinical outcomes observed in this subset of patients.

Moving from clinical and immunologic correlates to histopathological determinants, tumor morphology and subtype also play a key role in shaping immunotherapy outcomes. Our findings indicate that melanoma subtype exerts a notable influence on both response and survival outcomes under PD-1-based immunotherapy. Specifically, patients with acral melanoma exhibit significantly poorer overall survival compared with those with cutaneous melanoma, and this pattern remains consistent after adjustment for key clinical and pathological covariates, suggesting that the inferior outcomes observed in acral melanoma are not solely attributable to confounding factors such as disease stage or treatment exposure. UVA on OS for acral melanoma showed considerable heterogeneity. This heterogeneity may be attributed to varying definitions of acral melanoma across studies or differences in ethnicity between Asian and Western cohorts. The poorer clinical outcomes observed in acral melanoma appear to align with underlying biological differences, as this subtype exhibits reduced responsiveness to immune checkpoint blockade, likely driven by its distinct molecular and immunologic profile. Unlike cutaneous melanoma, acral melanoma arises in sun-shielded areas and therefore may lack the ultraviolet (UV)-induced mutational signature that drives high tumor mutational burden and neoantigen diversity. As a result, acral tumors have been shown to harbor fewer single-nucleotide variants and a predominance of structural and copy-number alterations, leading to reduced immunogenicity [108,109]. The tumor microenvironment in acral melanoma is further characterized by limited infiltration of cytotoxic CD8^+^ T cells and NK cells, producing a “cold” immune phenotype that restricts the efficacy of PD-1 blockade [110]. In addition, recurrent genomic alterations, including CDK4/CCND1 amplifications and NF2 deletions, have been linked to intrinsic resistance to anti-PD-1 therapy [105]. Collectively, these molecular and microenvironmental features might underlie the attenuated responsiveness and inferior survival outcomes associated with acral melanoma, thereby underscoring the need for subtype-specific therapeutic strategies and rational combinatorial approaches to improve clinical benefit in this distinct subgroup.

In addition to histologic subtype, disease stage prior to the initiation of immunotherapy is a significant determinant of treatment outcomes. Patients with more advanced disease demonstrated consistently poorer overall survival compared to those with lower-stage melanoma, both in multivariate and univariate models. The strength of this association was particularly robust in multivariate analyses, underscoring the independent prognostic value of stage even after adjustment for other clinical factors. Although univariate analyses also supported a negative impact of advanced stage, variability among studies suggests that differences in baseline characteristics, therapeutic regimens, and follow-up periods may partly account for the observed heterogeneity. Sensitivity analyses indicated that, while the direction of association remained consistent, effect estimates varied across studies, suggesting that large pooled cohorts may disproportionately influence the overall results. Beyond its statistical significance, the prognostic impact of disease stage likely reflects underlying biological mechanisms that limit immunotherapy efficacy in advanced melanoma. Importantly, patients with advanced-stage disease not only experience reduced overall survival but also lower response rates to immune checkpoint blockade compared with earlier-stage disease. This diminished efficacy is likely multifactorial: high tumor burden and visceral involvement restrict immune surveillance, while the advanced tumor microenvironment becomes profoundly immunosuppressive, characterized by elevated IL-6 levels, regulatory T-cell infiltration, and exhausted CD8^+^ T cells. Late-stage tumors also acquire immune escape mechanisms, including impaired interferon-γ signaling, MHC class I downregulation, and upregulation of alternative inhibitory checkpoints (TIM-3, VISTA, and LAG-3). Hypoxia within bulky lesions further compromises T-cell function [111,112,113]. Together, these data reinforce that disease stage not only defines disease extent but also mirrors biological resistance mechanisms that constrain immunotherapy efficacy.

Within the histopathological spectrum, tumor mutational burden (TMB) represents a key intrinsic characteristic of melanoma tissue that influences immunotherapy outcomes. In our meta-analysis, patients with metastatic melanoma and high tumor mutational burden (TMB) exhibited more favorable progression-free survival compared with those with low TMB following treatment with immune checkpoint inhibitors. This association was consistent across studies, with no indication of substantial heterogeneity, supporting the robustness of the observed relationship. Sensitivity analyses confirmed the stability of this effect across models. The biological basis for this finding is supported by previous evidence linking high TMB to enhanced sensitivity to immune checkpoint blockade through several plausible mechanisms. Tumors with an increased mutational load are more likely to generate a diverse repertoire of neoantigens capable of eliciting T-cell recognition and activation [114]. When these neoantigens are clonally expressed across tumor cells, they may promote more sustained antitumor immune surveillance. In some tumor types, elevated TMB also correlates with a more inflamed tumor microenvironment, characterized by increased CD8^+^ T-cell infiltration, interferon-γ signaling, and PD-L1 expression, which may facilitate immune engagement [115,116]. Furthermore, genomic features such as HLA heterozygosity and mutational diversity can broaden neoantigen presentation, potentially contributing to improved immune responsiveness [117,118]. Collectively, these mechanistic insights align with and may underlie the association observed in our analysis, suggesting that elevated TMB serves as a surrogate marker of increased tumor immunogenicity and enhanced sensitivity to immune checkpoint inhibition.

The genomic landscape of melanoma adds another critical dimension to the understanding of immunotherapy response. PD-L1 expression remains one of the most widely investigated histopathologic biomarkers in melanoma. In our meta-analysis of patients with metastatic melanoma, PD-L1 expression was not significantly associated with treatment outcomes. This lack of a clear relationship may result from both biological and methodological factors. Biologically, PD-L1 can exist in different forms and act at multiple levels. The soluble PD-L1 (sPD-L1) remains biologically active and can bind to PD-1 on T cells, leading to suppression of their function and promoting systemic immunosuppression. High circulating sPD-L1 levels have been associated with poor prognosis and may indicate a higher tumor burden or an exhausted immune system [119,120]. In contrast, tissue PD-L1 expression measured by immunohistochemistry may not fully reflect this systemic activity, and its evaluation is affected by heterogeneity in antibodies, scoring methods, and cutoff values used across studies. Moreover, PD-L1 behaves as a context-dependent molecule whose effects may vary according to expression levels and tumor environment. Similar to other cellular proteins such as SPD-L1, where moderate expression supports tumor progression while excessive expression induces detrimental genomic instability [121,122,123,124], PD-L1 might also exert different effects depending on its expression threshold. Moderate PD-L1 levels may facilitate immune escape, whereas very high levels could represent a reactive, but ineffective, immune activation. These biological complexities, combined with methodological variability and clinical heterogeneity across studies, likely explain why PD-L1 expression did not show a statistically significant impact on immunotherapy outcomes in our analysis and why published results remain inconsistent. To obtain more consistent evidence, future studies should adopt standardized PD-L1 assessment methods with harmonized assays and uniform cutoffs, while integrating longitudinal tissue and circulating PD-L1 measurements together with genomic immune markers to better capture the dynamic tumor–immune interplay and its impact on treatment response.

In the era of liquid biopsy, circulating biomarkers such as cell-free DNA and exosomal components provide a dynamic window into tumor–immune interactions. Among these, detectable or elevated circulating tumor DNA (ctDNA) prior to the start of immunotherapy was consistently associated with poorer clinical outcomes in patients with metastatic melanoma, including both overall survival and progression-free survival. The absence of significant heterogeneity across studies supports the reliability of this association in different therapeutic settings, including monotherapy and combination regimens. Sensitivity analyses confirmed the consistency of this association but revealed variability in effect magnitude across cohorts, likely reflecting differences in patient characteristics and ctDNA assessment methods. Biologically, these findings align with emerging evidence linking elevated ctDNA to adverse tumor–immune dynamics. High ctDNA levels reflect not only greater tumor burden but also an immunosuppressive microenvironment characterized by T-cell exhaustion, reduced cytotoxic infiltration, and enrichment of regulatory and myeloid-derived suppressor cells, all of which dampen responses to PD-1/PD-L1 inhibition. Furthermore, ctDNA positivity often denotes pronounced intratumoral heterogeneity, with subclonal mutations diminishing neoantigen uniformity and immune recognition [125]. Recurrent genomic alterations identified in ctDNA, such as PTEN loss, STK11 mutations, β-catenin activation, and JAK1/2 or B2M inactivation, have been associated with primary and acquired resistance to checkpoint blockade [126,127]. Longitudinal analyses further demonstrate that persistent or rising ctDNA during treatment predicts early progression and therapeutic failure [128,129]. Collectively, these data support ctDNA as a dynamic biomarker integrating tumor burden, genomic instability, and immune evasion—offering a mechanistic rationale for its consistent association with reduced immunotherapy efficacy and poorer survival outcomes.

Despite the strengths of this comprehensive synthesis, certain limitations should be acknowledged. Formal assessment of publication bias (e.g., funnel plots) was not performed due to the limited number of studies (<10) available for most individual predictors. Further, moderate-to-high heterogeneity between estimates for some outcomes may influence our results. To address this issue, several sensitivity analyses and subgroup explorations were conducted to identify potential sources of heterogeneity and to assess the robustness of pooled estimates. The variability in statistical adjustment and reporting standards across cohorts may also have influenced pooled estimates, particularly for factors such as PD-L1 expression, and ctDNA, where analytical methodologies differed considerably. Finally, the continuous evolution of therapeutic strategies—including combination and neoadjuvant immunotherapy—means that some data reflect earlier treatment eras and may not fully represent current clinical practice. Taken together, these considerations highlight both the inherent challenges of meta-analytic synthesis in a rapidly evolving field and the need for large, prospective, harmonized datasets to validate and refine the prognostic relevance of the identified factors.

In conclusion, this meta-analysis provides a comprehensive synthesis of clinicopathologic, laboratory, and histopathologic determinants of immunotherapy outcomes in metastatic melanoma. Performance status, age, LDH levels, BMI, and metastatic burden emerged as consistent clinical and biochemical predictors, while tumor ulceration, disease stage, and TMB were among the most robust histologic correlates of prognosis. Conversely, PD-L1 expression and gender did not demonstrate consistent predictive value, whereas the occurrence of cutaneous immune-related adverse events and elevated BMI were associated with favorable outcomes. Moreover, ctDNA levels and brain metastases consistently reflected poor prognosis, emphasizing their potential as dynamic and clinically actionable biomarkers. Together, these insights reinforce the need for multidimensional prognostic models that transcend single biomarkers and capture the complex interplay between tumor biology, host immunity, and treatment dynamics.

## 5. Future Directions

Prospective biomarker-driven trials are needed to validate integrated predictive models that combine clinical, biochemical, histological, and genomic markers. Exploration of dynamic biomarkers such as circulating tumor DNA (ctDNA), cytokine profiles, immune phenotyping, or T-cell receptor (TCR) clonality may enable real-time monitoring of therapy response. Rigorous molecular analysis of PD-L1, TMB, and TILs—standardizing assays across institutions—could unlock new predictors. Investigating sequencing strategies (e.g., ICI before or after BRAF/MEK inhibitors) through high-quality comparative trials.

## Figures and Tables

**Table 1:** Studies included in the systematic review.

ID	Author/Year	Total Number of Patients/Arm	Response Outcome	Within Arm Predictors Examined	Outcomes	Results
1	Majidova et al. [27]	BRAFi + MEKi **44** PD-L1i **30** PD-L1i + CTLA4i **14** CTLA4i **6** CΤ **26**	BRAFi + MEKi CR **6** PR **18** SD **14** PD **6** ICI CR **8** PR **17** SD **9** PD **14** CT CR **8** PR **17** SD **9** PD **14**	Ulceration	OS	BRAF + MEKi/PD-L1i +/- CTLA4i/CT (UVA-OS) Ulceration (yes vs. no) **(HR 1.85, 95% CI: 1.03 to 3.33, *p* = 0.039)**
2	Martinez-Recio et al. [70]	PD-L1i **214** PD-L1i + CTLA4i **31**	-	Subtype Ulceration	OS	PD-L1i +/- CTLA4i (MVA-OS) Subtype (acral vs. cutaneous) **(HR 3.9, 95% CI: 1.37 to 11.11, *p* = 0.01)** PD-L1i +/- CTLA4i (UVA-OS) Subtype (mucosal vs. cutaneous) **(HR 3.58, 95% CI: 1.27 to 10.1, *p* = 0.02)** Ulceration (yes vs. no) **(HR 1.96, 95% CI: 1.01 to 3.77, *p* = 0.04)**
3	Pedersen et al. [17]	BRAF/MEKi **235** PD-L1i **189** PD-L1i + CTLA4i **161** CTLA4i **30**	-	ECOG LDH	OS	BRAF + MEKi/PD-L1i +/- CTLA4i (MVA-OS) ECOG (≥2 vs. <2) **(HR 1.90, 95% CI: 1.55 to 2.32, *p* < 0.0001)** LDH (elevated vs. normal) **(HR 1.58, 95% CI: 1.32 to 1.89, *p* < 0.0001)**
4	Zhang et al. [20]	PD-L1i **94**	CR **0** PR **7** SD **55** PD **32**	ECOG No metastatic sites	OS	PD-L1i (UVA-OS) ECOG (≥2 vs. <2) **(HR 2.43, 95% CI: 1.35 to 4.34, *p* = 0.003)** No metastatic sites (≥3 vs. <3) **(HR 2.36, 95% CI: 1.35 to 4.1, *p* = 0.003)**
5	Jansen et al. [14]	CTLA4i + PD-L1i **148** PDLi1 **129** BRAFi **97** PD-L1i + et al. **7** Chemo **9** Et al **2**	-	Subtype Age	OS PFS	CTLA4i + PD-L1i (UVA-OS) Subtype (mucosal vs. cutaneous) **(HR: 2.96, 95% CI: 1.65 to 5.31 *p* < 0.001)** Age (≥65 vs. <65) **(HR: 2.56, 95% CI: 1.56 to 4.20 *p* < 0.001)** PD-L1i (UVA-OS) Age (≥65 vs. <65) **(HR: 2.08, 95% CI: 1.13 to 3.84, *p* = 0.02)** PD-L1i (UVA-PFS) Age (≥65 vs. <65) **(HR: 2.56, 95% CI: 1.56 to 4.20, *p* = 0.0002)**
6	Derks et al. [24]	PD-L1i + CTLA4i **40** PD-L1i **39** CTLA4i **3**	Total CR **12** PR **27** SD **9** PD **34**	ECOG Age	OS	PD-L1i + CTLA4i/CTLA4i/PD-L1i (MVA-OS) Age (continuous) **(HR 1.03, 95% CI: 1.01 to 1.06, *p* = 0.006)** PD-L1i + CTLA4i/CTLA4i/PD-L1i (UVA-OS) ECOG (≥2 vs. <2) **(HR 2.32, 95% CI: 1.03 to 5.22, *p* = 0.041)**
7	Kott et al. [15]	PD-L1i **1400** PD-L1i + CTLA4i **1019**	CR **255** PR **366** SD **314** PD **923** Νot **50** Unknown **511**	BM ECOG	OS PFS	PD-L1i +/- CTLA4i (MVA-OS) BM (yes vs. no) **(HR 1.30, 95% CI: 1.10 to 1.60 *p* < 0.001)** ECOG (≥1 vs. 0) **(HR 3.20, 95% CI: 2.40 to 4.10 *p* < 0.001)** PD-L1i +/- CTLA4i (MVA-PFS) BM (yes vs. no) **(HR 1.20, 95% CI: 1.10 to 1.40 *p* = 0.016)** ECOG (≥1 vs. 0) **(HR 1.90, 95% CI: 1.42 to 2.54 *p* < 0.001)** PD-L1i +/- CTLA4i (UVA-OS) BM (yes vs. no) **(HR 1.30, 95% CI: 1.1 to 1.6 *p* < 0.001)** ECOG (≥2 vs. <2) **(HR 3.2, 95% CI: 2.4 to 4.2, *p* < 0.001)** PD-L1i +/- CTLA4i (UVA-PFS) ECOG (≥1 vs. 0) **(HR 1.90, 95% CI: 1.42 to 2.54 *p* < 0.001)**
8	Verkhovskaia et al. [58]	CTLA4i **44**	Total CR **1** PR **10** SD **2** PD **30** Unknown **1**	BM	OS	CTLA4i (MVA-OS) BM (yes vs. no) **(HR 4.60, 95% CI: 1.10 to 19.2, *p* = 0.037)** CTLA4i (UVA-OS) BM (yes vs. no) **(HR 4.50, 95% CI: 1.03 to 19.1, *p* = 0.037)**
9	Silasi et al. [42]	PD-L1i + CTLA4i **53**	-	ECOG LDH	OS PFS	PD-L1i + CTLA4i (MVA-OS) ECOG (≥1 vs. 0) **(HR 6.25, 95% CI: 1.19 to 33.33, *p* = 0.031)** LDH (elevated vs. normal) **(HR 2.90, 95% CI: 1.11 to 7.55, *p* = 0.03)** PD-L1i + CTLA4i (UVA-OS) ECOG (≥1 vs. 0) **(HR 4.55, 95% CI: 1.08 to 20, *p* = 0.038)** PD-L1i + CTLA4i (UVA-PFS) ECOG (≥1 vs. 0) **(HR 4.76, 95% CI: 1.12 to 20, *p* = 0.033)**
10	Mandala et al. [16]	PD-L1i + CTLA4i **376**	CR **49** PR **109** SD **33** PD **159** Unknown **21**	ECOG LDH	OS	PD-L1i + CTLA4i (MVA-OS) ECOG (≥1 vs. 0) **(HR 1.97, 95% CI: 1.46 to 2.66, *p* < 0.001)** PD-L1i + CTLA4i (UVA-OS) ECOG (≥1 vs. 0) **(HR 2.34, 95% CI: 1.77 to 3.11)** LDH (elevated vs. normal) **(HR 1.34, 95% CI: 1.01 to 1.78)**
11	Chen et al. [32]	PD-L1i **38** PD-L1i + CTLA4i **5**	CR **4** PR **15** SD **19** PD **3** Unknown **2**	Age LDH	OS	PD-L1i +/- CTLA4i (MVA-OS) LDH (elevated vs. normal) **(HR 2.46, 95% CI: 1.21 to 5.00, *p* = 0.013)** PD-L1i +/- CTLA4i (UVA-OS) Age (≥65 vs. <65) **(HR 2.07, 95% CI: 1.07 to 3.98, *p* = 0.029)** LDH (elevated vs. normal) **(HR 3.025, 95% CI: 1.53 to 5.98, *p* = 0.001)**
12	Kopecký et al. [40]	PD-L1i **65**	PD-L1i CR **17** PR **16** SD **14** PD **18**	LHD Liver metastasis	PFS	PD-L1i (MVA-PFS) LDH (elevated vs. normal) **(HR 2.24, 95% CI: 1.11 to 4.53, *p* = 0.02)** ΤΤ (MVA-PFS) LDH (elevated vs. normal) **(HR 1.75, 95% CI: 1.08 to 2.77, *p* = 0.02)** PD-L1i (UVA-PFS) LDH (elevated vs. normal) **(HR 1.87, 95% CI: 1.30 to 2.71, *p* = 0.0008)** Liver metastasis (yes vs. no) **(HR 1.68, 95% CI: 1.12 to 2.54, *p* = 0.013)**
13	Sun et al. [19]	PD-L1i + CTLA4i **36** PD-L1i **64** BRAFi + MEKi **50**	PD-L1i + CTLA4i CR **4** PR **10** SD **14** PD **8** PD-L1i CR **12** PR **24** SD **7** PD **21** BRAFi + MEKi CR **8** PR **27** SD **4** PD **11**	Age ECOG BM Liver metastasis LDH	OS PFS	BRAF + MEKi/PD-L1i +/- CTLA4i (MVA-OS) ECOG (≥2 vs. <2) **(HR 2.58, 95% CI: 1.38 to 3.54 *p* < 0.01)** BM (yes vs. no) **(HR 1.78, 95% CI: 1.25 to 3.33 *p* < 0.01)** LDH (elevated vs. normal) **(HR 1.82, 95% CI: 1.14 to 2.7, *p* = 0.03)** BRAF + MEKi/PD-L1i +/- CTLA4i (MVA-PFS) ECOG (≥2 vs. <2) **(HR 2.01, 95% CI: 1.99 to 3.13 *p* = 0.02)** BM (yes vs. no) **(HR 2.14, 95% CI: 1.28 to 3.56 *p* = 0.02)** LDH (elevated vs. normal) **(HR 1.61, 95% CI: 1.12 to 3.57, *p* = 0.03)** BM (yes vs. no) **(HR 2.14, 95% CI: 1.28 to 3.56 *p* < 0.02)** BRAF + MEKi/PD-L1i +/- CTLA4i (UVA-OS) Age (≥65 vs. <65) **(HR 1.67, 95% CI: 1.37 to 1.93 *p* = 0.03)** ECOG (≥2 vs. <2) **(HR 2.43, 95% CI: 1.87 to 3.34 *p* = 0.03)** BM (yes vs. no) **(HR 2.07, 95% CI: 1.29 to 3.32 *p* < 0.01)** BRAF + MEKi/PD-L1i +/- CTLA4i (UVA-PFS) Age (≥65 vs. <65) **(HR 1.91, 95% CI: 1.15 to 3.02 *p* = 0.04)** ECOG (≥2 vs. <2) **(HR 2.15, 95% CI: 1.95 to 3.08 *p* = 0.02)** BM (yes vs. no) **(HR 1.55, 95% CI: 0.90 to 2.68 *p* < 0.03)** Liver metastasis (yes vs. no) **(HR 1.55, 95% CI: 0.9 to 2.68 *p* = 0.03)** LDH (elevated vs. normal) **(HR 1.66, 95% CI: 0.7 to 3.84, *p* = 0.03)**
14	da Silva et al. [18]	PD-L1i **284** PD-L1i + CTLA4i **249**	PD-L1 CR **40** PR **60** SD **40** PD **145** PD-L1i + CTLA4i CR **32** PR **82** SD **27** PD **105**	Subtype ECOG LDH	OS PFS	PD-L1i +/- CTLA4i (MVA-OS) ECOG (≥1 vs. 0) **(HR 1.69, 95% CI: 1.30 to 2.21 *p* < 0.001)** LDH (elevated vs. normal) **(HR 1.50, 95% CI: 1.15 to 1.96 *p* = 0.003)** Subtype (acral vs. cutaneous) **(HR 2.96, 95% CI: 2.47 to 3.59 *p* < 0.004)** PD-L1i +/- CTLA4i (MVA-PFS) ECOG (≥1 vs. 0) **(HR 1.47, 95% CI: 1.17 to 1.85 *p* < 0.001)** LDH (elevated vs. normal) **(HR 1.33, 95% CI: 1.06 to 1.67 *p* = 0.015)**
15	Jan et al. [75]	PD-L1i **1218** CTLA4i **195** PD-L1i + CTLA4i **635** BRAFi + MEKi **52**	-	BMI	OS	BRAF + MEKi/PD-L1i +/- CTLA4i (UVA-OS) BMI (≥25 kg/m^2^ vs. <25 kg/m^2^) **(HR 0.68, 95% CI: 0.60 to 0.79 *p* < 0.001)**
16	Zhao et al. [55]	PD-L1i + IFN + TKi **55**	CR **1** PR **4** SD **21** PD **29**	Stage Skin disorder	OS PFS	PD-L1i + IFN + TKi (UVA-OS): Stage (IV vs. ≤III) **(HR 7.69, 95% CI: 1.66 to 33.33 *p* = 0.009)** Skin disorder (no vs. yes) **(HR 0.34, 95% CI: 0.13 to 0.89 *p* = 0.029)** PD-L1i + IFN + TKi (UVA-PFS): Skin disorder (no vs. yes) **(HR 0.45, 95% CI: 0.21 to 0.93 *p* = 0.033)**
17	Long et al. [44]	PD-L1i + CTLA4i **839** PD-L1i **536**	-	AGE ECOG LDH Stage Liver metastasis	OS	PD-L1i + CTLA4i (MVA-OS) ECOG (≥1 vs. 0) **(HR 1.59, 95% CI: 1.26 to1.99 *p* < 0.001)** PD-L1i (MVA-OS) ECOG (≥1 vs. 0) **(HR: 1.91, 95% CI: 1.45 to 2.50 *p* < 0.001)** Liver metastasis (yes vs. no) **(HR: 1.55, 95% CI: 1.15 to 2.09 *p* < 0.004)** PD-L1i (UVA-OS) Stage (IV vs. ≤III) **(HR: 1.85, 95% CI: 1.44 to 2.32 *p* < 0.001)** Liver metastasis (yes vs. no) LDH (elevated vs. normal) **(HR 2.12, 95% CI: 1.69 to 2.65 *p* = 0.0004)** **(HR: 1.47, 95% CI: 1.2 to 1.8 *p* < 0.001)** PD-L1i + CTLA4i (UVA-OS) Age (≥65 vs. <65) **(HR: 1.35, 95% CI: 1.11 to 1.66 *p* < 0.003)** ECOG (≥1 vs. 0) **(HR: 1.81, 95% CI: 1.46 to 2.25 *p* < 0.001)** Stage (IV vs. ≤III) **(HR: 1.59, 95% CI: 1.28 to 1.92 *p* < 0.001)** Liver metastasis (yes vs. no) **(HR: 1.47, 95% CI: 1.2 to 1.80 *p* < 0.001)** LDH (elevated vs. normal) **(HR 1.99, 95% CI: 1.63 to 2.44 *p* = 0.001)**
18	Marchisio et al. [28]	BRAFi + MEKi **28** PD-L1i **3**	-	ctDNA Age Stage	OS	PD-L1i/BRAFi + MEKi (MVA-OS) Age (continuous) **(HR: 1.07 95% CI: 1.01 to 1.14 *p* = 0.015)** PD-L1i/BRAFi + MEKi (UVA-OS) Stage (IV vs. ≤III) **(HR: 8.81 95% CI: 1.68 to 46.21 *p* = 0.01)** ctDNA status (high vs. low) **(HR: 7.92 95% CI: 1.56 to 40.36 *p* = 0.013)**
19	Rozendorn et al. [41]	PD-L1i **86** CTLA4i **6** PD-L1i + CTLA4i **31** BRAFi + MEKi **15**	-	LDH	OS	BRAF + MEKi/PD-L1i +/- CTLA4i (ΜVA-OS) LDH (elevated vs. normal) **(HR 3.04, 95% CI: 1.77 to 5.24 *p* < 0.001)** BRAF + MEKi/PD-L1i +/- CTLA4i (UVA-OS) LDH (elevated vs. normal) **(HR 3.04, 95% CI: 1.77 to 5.24 *p* < 0.001)**
20	Roccuzzo et al. [57]	PD-L1i (Nivo) **63** PD-L1i (Pem) **19** BRAFi + MEKi **82**	-	Age Ulceration Skin disorder	OS PFS	PD-L1i/BRAFi + MEKi (MVA-OS) Age (continuous) **(HR: 1.04 95% CI: 1.01 to 1.08 *p* = 0.015)** PD-L1i/BRAFi + MEKi (UVA-OS) Ulceration (yes vs. no) **(HR 2.80, 95% CI: 1.26 to 6.22, *p* = 0.011)** Skin disorder (no vs. yes) **(HR 0.21, 95% CI: 0.07 to 0.61, *p* < 0.001)** PD-L1i/BRAFi + MEKi (UVA-PFS) Ulceration (yes vs. no) **(HR 4.57, 95% CI: 2.27 to 9.11, *p* = 0.001)**
21	Cybulska-Stopa et al. [36]	PD-L1i **455**	PD-L1i CR **27** PR **121** SD **125** PD **165** Unknown **17**	Age ECOG BM LDH	OS PFS	PD-L1i (MVA-OS) Age (≥80 vs. <80) **(HR 1.40, 95% CI: 1.02 to 1.93, *p* < 0.0241)** BM (yes vs. no) **(HR 1.42, 95% CI: 1.11 to 2, *p* = 0.0088)** LDH (elevated vs. normal) **(HR 1.72, 95% CI: 1.45 to 2.04, *p* < 0.0001)** ECOG (≥1 vs. 0) **(HR 3.84, 95% CI: 1.66 to 10, *p* < 0.0001)** PD-L1i (MVA-PFS) BM (yes vs. no) **(HR 1.43, 95% CI: 1.11 to 2, *p* = 0.008)** LDH (elevated vs. normal) **(HR 1.72, 95% CI: 1.42 to 2.03, *p* < 0.0001)** ECOG (≥1 vs. 0) **(HR 3.45, 95% CI: 1.7 to 10, *p* < 0.0001)** PD-L1i (UVA-OS) BM (yes vs. no) **(HR 2.08, 95% CI: 1.43 to 3.33, *p* < 0.0001)** LDH (elevated vs. normal) **(HR 2.17, 95% CI: 1.66 to 3.33, *p* < 0.0001)** ECOG (≥1 vs. 0) **(HR 1.85, 95% CI: 1.43 to 2.45, *p* < 0.0001)** PD-L1i (UVA-PFS) BM (yes vs. no) **(HR 1.56, 95% CI: 1.25 to 2.00, *p* < 0.0003)** LDH (elevated vs. normal) **(HR 1.75, 95% CI: 1.43 to 2.07, *p* < 0.0001)** ECOG (≥1 vs. 0) **(HR 1.66, 95% CI: 1.11 to 3.33, *p* < 0.046)**
22	Internò et al. [39]	PD-L1i **44** BRAFi + MEKi **43**	-	LDH	OS	PD-L1i/BRAFi + MEKi (MVA-OS) LDH (elevated vs. normal) **(HR 1.85, 95% CI: 0.96 to 3.56 *p* = 0.045)** PD-L1i/BRAFi + MEKi (UVA-OS) LDH (elevated vs. normal) **(HR 2.58, 95% CI: 1.41 to 4.74 *p* = 0.002)**
23	Haist et al. [48]	PD-L1i **152** BRAFi + MEKi **88**	-	Ulceration BM Liver metastasis No metastatic sites	OS PFS	PD-L1i/BRAFi + MEKi (MVA-OS) No metastatic sites (>2 vs. ≤2) **(HR 3.19, 95% CI: 1.28 to 7.99, *p* = 0.013)** PD-L1i/BRAFi + MEKi (UVA-OS) BM (yes vs. no) **(HR 1.98, 95% CI: 1.14 to 3.42, *p* = 0.015)** PD-L1i/BRAFi + MEKi (UVA-PFS) Ulceration (yes vs. no) **(HR 2.10, 95% CI: 1.31 to 3.36, *p* = 0.002)** BM (yes vs. no) **(HR 1.60, 95% CI: 1.03 to 2.48, *p* = 0.037)** Liver metastasis (yes vs. no) **(HR 2.11, 95% CI: 1.34 to 3.31, *p* = 0.001)**
24	Namikawa et al. [56]	BRAF/MEKi **236** PD-L1i **64** CTLA4 (IPI) + PD-L1i (NIVO) **36**	BRAF/MEKi CR **49** PR **114** SD **36** PD **22** PD-L1i CR **13** PR **4** SD **16** PD **26** PD-L1i/CTLA4i CR **2** PR **8** SD **8** PD **13**	ECOG No metastatic sites LDH	OS PFS	BRAF + MEKi/PD-L1i +/- CTLA4i (MVA-OS) ECOG (≥2 vs. <2) **(HR 3.57, 95% CI: 2.08 to 6.12, *p* < 0.001)** LDH (elevated vs. normal) **(HR 2.01, 95% CI: 1.48 to 2.72, *p* < 0.001)** BRAF + MEKi/PD-L1i +/- CTLA4i (MVA-PFS) ECOG (≥2 vs. <2) **(HR 2.49, 95% CI: 1.54 to 4.03, *p* < 0.001)** LDH (elevated vs. normal) **(HR 2.01, 95% CI: 1.49 to 2.73, *p* < 0.001)** BRAF + MEKi/PD-L1i +/- CTLA4i (UVA-OS) ECOG (≥2 vs. <2) **(HR 4.45, 95% CI: 2.70 to 7.35, *p* < 0.001)** No metastatic sites (≥3 vs. <3) **(HR 2.42, 95% CI: 1.71 to 2.98, *p* < 0.001)** LDH (elevated vs. normal) **(HR 2.26, 95% CI: 1.71 to 2.98, *p* < 0.001)** BRAF + MEKi/PD-L1i +/- CTLA4i (UVA-PFS) ECOG (≥2 vs. <2) **(HR 2.64, 95% CI: 1.68 to 4.15, *p* < 0.001)**
25	Afrăsânie et al. [37]	PDLi1 (Nivo) **51**	-	Liver metastasis	OS	PD-L1i (OS-MVA) Liver metastasis (yes vs. no) **(HR: 4.42, 95% CI: 1.88 to 10.4 *p* = 0.001)** PD-L1i (OS-UVA) Liver metastasis (yes vs. no) **(HR: 3.5, 95% CI: 1.59 to 7.69, *p* = 0.002)**
26	Franklin et al. [38]	PD-L1i **808** CTLA4i **134** PD-L1i + CTLA4i **389** BRAFi + MEKi **373**	CR **211** PR **941** SD **215** PD **602** Unknown **382**	LDH Stage	**OS**	**BRAF + MEKi/PD-L1i +/- CTLA4i (ΜVA-OS)** **LDH (elevated vs. normal)** **(HR: 1.43, 95% CI: 1.11 to 1.85 *p* = 0.006)** **Stage (IV vs. ≤III)** **(HR: 1.61, 95% CI: 1.16 to 2.22 *p* = 0.004)** **BRAF + MEKi/PD-L1i +/- CTLA4i (UVA-OS)** **LDH (elevated vs. normal)** **(HR 1.45, 95% CI: 1.16 to 1.81, *p* < 0.001)**
27	Rousset et al. [25]	PD-L1i **102** CTLA4i **21** PD-L1i + CTLA4i **42**	CR **31** PR **36** SD **46** PD **5**	Stage ECOG LDH BM Liver metastasis	OS PFS	PD-L1i +/- CTLA4i (MVA-OS) Stage (IV vs. ≤III) **(HR 1.53, 95% CI: 1.28 to 1.83 *p* < 0.001)** ECOG (≥1 vs. 0) **(HR 1.76, 95% CI: 1.54 to 2.01 *p* < 0.001)** LDH (elevated vs. normal) **(HR 1.51, 95% CI: 1.31 to 1.78 *p* < 0.001)** Liver metastasis (yes vs. no) **(HR 1.31, 95% CI: 1.1 to 1.56 *p* = 0.01)** BM (yes vs. no) **(HR 1.4, 95% CI: 1.19 to 1.65, *p* = 0.001)** PD-L1i +/- CTLA4i (MVA-PFS) ECOG (≥1 vs. 0) **(HR 1.44, 95% CI: 1.29 to 1.62 *p* < 0.001)** LDH (elevated vs. normal) **(HR 1.37, 95% CI: 1.21 to 1.55 *p* < 0.001)** BM (yes vs. no) **(HR 1.27, 95% CI: 1.11 to 1.46, *p* = 0.001)**
28	Kartolo et al. [26]	PD-L1i **80** PD-L1i + CTLA4i **16**	CR **10** PR **18** SD **10** PD **58**	ECOG No metastatic sites	OS	PD-L1i +/- CTLA4i (MVA-OS) ECOG (>2 vs. ≤2) **(HR 2.30, 95% CI: 1.08 to 4.85 *p* = 0.029)** No metastatic sites (>2 vs. ≤2) **(HR 2.04, 95% CI: 1.19 to 3.49 *p* = 0.009)**
29	Di Nardo et al. [43]	PD-L1i **28** PD-L1i + CTLA4i **1** BRAFi/MEKi **19**	-	Circulating tumor DNA (ctDNA)	OS PFS	PD-L1i/PD-L1i + CTLA4i/BRFAi-MEKi (MVA-OS) ctDNA (high vs. low) **(HR 4.39, 95% CI: 1.38 to 13.97,** ***p* = 0.012)** PD-L1i/PD-L1i + CTLA4i/BRFAi-MEKi (MVA-PFS) ctDNA (high vs. low) **(HR 3.65, 95% CI: 1.08 to 10.93,** ***p* = 0.021)**
30	Carvajal et al. [52]	ImmTAC **42**	CR **0** PR **5** SD **9** PD **28**	Skin disorder Circulating tumor DNA (ctDNA)	OS	ImmTAC (UVA-OS) Skin disorder (no vs. yes) **(HR 0.235, 95% CI: 0.10 to 0.54, *p* < 0.001)** ctDNA (high vs. low) **(HR 4.35, 95% CI: 2.43 to 7.7, *p* < 0.001)**
31	Mattila et al. [51]	BRAFi + CT **19**	-	Circulating tumor DNA (ctDNA)	OS PFS	BRAFi + CT (MVA-OS) ctDNA (high vs. low) **(HR 14.6, 95% CI: 2.24 to 95.46,** ***p* = 0.005)** BRAFi + CT (MVA-PFS) ctDNA (high vs. low) **(HR 6.1, 95% CI: 1.39 to 24.57,** ***p* = 0.012)**
32	Howell et al. [53]	PD-L1i +/- CTLA4i (**1435**)	-	Age No metastatic sites	OS	PD-L1i +/- CTLA4i (MVA-OS) Age (≥80 vs. <80) **(HR 1.24, 95%CI: 1.15 to 1.32, *p* = 0.01)** No metastatic sites (>2 vs. ≤2) **(HR 2.37, 95%CI: 2.08 to 2.71, *p* = 0.01)**
33	Kim et al. [33]	PD-L1i/ATR **29**	CR **0** PR **9** SD **10** PD **10**	BM	OS PFS	PD-L1i/ATR (UVA-OS) BM (yes vs. no) **(HR 6.10, 95% CI: 1.60 to 23.28, *p* = 0.008)** PD-L1i/ATR (UVA-PFS) BM (yes vs. no) **(HR 2.45, 95% CI: 0.79 to 7.62, *p* = 0.012)**
34	Lee et al. [72]	Anti-PD-1/CTLA-4 **266**		BMI	OS	PD-L1i/CTLA4i (MVA-OS) BMI (≥25 kg/m^2^ vs. <25 kg/m^2^) **(HR 0.6, 95% CI: 037 to 0.99 *p* < 0.001)**
35	Nardin et al. [34]	CTLA4i **21** PD1i **36** PD-L1i + CTLA4i **1**	CR + PR **21** SD + PD **19** UN **12**	LDH	PFS	CTLA4i (UVA-PFS) LDH (elevated vs. normal) **(HR 1.99% CI: 1.01 to 3.95, *p* = 0.047)**
36	Yamada et al. [62]	PD1i **93**, CTLA4i **27**, PD1i + CTLA4i **10**	-	ECOG	OS	PD1i, CTLA4i, PD1i + CTLA4i (MVA-OS) ECOG (≥2 vs. <2) **(HR 2.41, 95% CI: 1.13 to 5.15), *p* = 0.024)**
37	Hodi et al. [49]	**NCT01721772:** PD1i **52** **NCT01844505:** TMB evaluable patients: PD1i **176** PD1i + CTLA4i **184** CTLA4i **178** Inflammatory signature (GEP) evaluable patients: PD1i **97** PD1i + CTLA4i **85** CTLA4i **87**	**NCT01721772:** CR 12 PR 17 SD 15 PD 8 **NCT01844505:** TMB evaluable patients: **PD1i** CR 24 PR 54 SD 53 PD 45 **PD1i + CTLA4i** CR 43 PR 57 SD 49 PD 35 **CTLA4i** CR 6 PR 24 SD 60 PD 88 Inflammatory signature (GEP) evaluable patients: **PD1i** CR 15 PR 27 SD 33 PD 22 **PD1i + CTLA4i** CR 23 PR 26 SD 19 PD 17 **CTLA4i** CR 5 PR 11 SD 33 PD 38	TMB	PFS	PD1i (067) (UVA-PFS) TMB (low vs. high) **(HR 0.34, 95% CI: 0.16 to 0.72)** PD1i + CTLA4i (067) (UVA-PFS) TMB (low vs. high) **HR 0.55, 95% CI: 0.38 to 0.81)** CTLA4i (067) (UVA-PFS) TMB (low vs. high) **(HR 0.60, 95% CI: 0.43 to 0.82)**
38	Zhou et al. [21]	PD1i (acral and mucosal) **114**	CR + PR **14** SD **42** PD **58**	ECOG LDH	OS PFS	PD1i (MVA-OS) ECOG (≥1 vs. 0) **(HR 1.93, 95% CI 1.19 to 3.14, *p* = 0.008)** LDH (elevated vs. normal) **(HR 2.23, 95% CI 1.43 to 3.49, *p* < 0.001)** PD1i (MVA-PFS) ECOG (≥1 vs. 0) **(HR 1.53, 95% CI 1.01 to 2.33, *p* = 0.048)** LDH (elevated vs. normal) **(HR 1.99, 95% CI 1.32 to 3.00, *p* = 0.001)**
39	Yamazaki et al. [47]	PD1i **124**	CR **3** PR **19** SD **29** PD **58** Unknown **15**	Skin disorder	PFS	PD1i (UVA-PFS) Skin disorder (no vs. yes) **(HR 0.52, 95% CI: 0.33 to 0.83, *p* = 0.006)**
40	Herbreteau et al. [77]	PD-L1i **93** PD-L1i + CTLA4i **9**	-	Circulating tumor DNA (ctDNA)	OS PFS	PD-L1i/PD-L1i + CTLA4i (MVA-OS) ctDNA (high vs. low) **(HR 10.52, 95% CI: 1.83 to 60.55,** ***p* = 0.008)** PD-L1i/PD-L1i + CTLA4i (MVA-PFS) ctDNA (high vs. low) **(HR 5.52, 95% CI: 1.22 to 24.90,** ***p* = 0.008)**
41	Di Filippo et al. [69]	PD-L1i+ CTLA4i **1214**	-	No metastatic sites BMI	OS	PD-L1i + CTLA4i (UVA-OS) No metastatic sites (≥3 vs. <3) **(HR 1.72, 95% CI: 1.39 to 2.13, *p* = 0.001)** BMI (≥25 kg/m^2^ vs. <25 kg/m^2^) **(HR 0.98, 95% CI: 0.75 to 1.28, *p* < 0.001**
42	Cowey et al. [60]	PD1i **81** BRAF/MEKi **143**	-	BM LDH	OS	PD1i/BRAFi/MEKi (MVA-OS) BM (yes vs. no) **(HR 2.19, 95%CI: 1.45 to 3.31, *p* = 0.0002)** LDH (elevated vs. normal) **(HR 2.15, 95%CI: 1.33 to 3.47, *p* = 0.0019)** PD1i/BRAFi/MEKi (UVA-OS) Age (≥65 vs. <65) **(HR: 1.79, 95% CI: 1.22 to 2.63 *p* < 0.0027)** BM (yes vs. no) **(HR 1.75, 95%CI: 1.18 to 2.6, *p* = 0.0049)** LDH (elevated vs. normal) **(HR 2.1, 95%CI: 1.28 to 3.45, *p* = 0.002)**
43	Săftescu et al. [35]	PD1i **42**	-	Liver metastasis	PFS	PD1i (MVA–PFS) LDH (elevated vs. normal) **(HR 1.43, 95% CI: 1.26 to 1.63, *p* < 0.0001)** ECOG (≥1 vs. 0) **(HR 1.2, 95% CI: 1.03 to 1.36, *p* = 0.0043)** PD1i (UVA-PFS) Liver metastasis (yes vs. no) **(HR 3.91, 95% CI: 1.41 to 10.85, *p* = 0.009)**
44	Puzanov et al. [45]	PD1i **1558**	-	LDH ECOG	OS PFS	
45	Young et al. [76]	Anti-PD-1 ± CTLA-4 **287**		BMI	OS	PD-L1i/CTLA4i (UVA-OS) BMI (≥25 kg/m^2^ vs. <25 kg/m^2^) **(HR 1.10, 95% CI: 0.71 to 1.70 *p* < 0.001)**
46	Yamazaki et al. [46]	CTL4i **547**	-	Subtype	OS	CTL4i (UVA-OS) Subtype (mucosal vs. cutaneous) **(HR 1.30, 95% CI: 1.05 to 1.60, *p* = 0.005)**
47	Lee et al. [71]	PD-L1i **42** PD-L1i + CTLA4i **30**	-	Circulating tumor DNA (ctDNA)	OS	PD-L1i/PD-L1i + CTLA4i (MVA-OS) ctDNA (high vs. low) **(HR 1.18, 95% CI: 1.06 to 3.57,** ***p* = 0.03)** BMI (≥25 kg/m vs. <25 kg/m^2^) **(HR 0.6, 95% Cl: 0.37 to 0.98)**
48	Donnelly et al. [74]	PD-L1i +/- CTLA4i **423**	-	Stage BMI	OS	PD-L1i/PD-L1i + CTLA4i (MVA-OS) Stage (IV vs. ≤III) **(HR 3.00, 95% CI: 1.07 to 8.41,** ***p* = 0.04)** BMI (≥25 kg/m vs. <25 kg/m^2^) **(HR 1.05, 95% Cl: 0.67 to 1.65)**
49	Ratnayake et al. [30]	SABR + PD1i/CTLA4i PD1i+CTLA4i **24**		Age	OS	PD1i/CTLA4i/PD1i + CTLA4i (MVA-OS) Age (continuous) **HR 1.18, 95% CI: 1.05 to 1.33 ( *p* < 0.01)**
50	Rutkowski et al. [73]	BRAFi ± MEKi CTLA4i + PD1i **668**		BMI	OS	BRAFi ± MEKi/CTLA4i + PD1i (MVA-OS) BMI (≥25 kg/m^2^ vs. <25 kg/m^2^) **(HR 1.09, 95% CI: 0.79 to 1.5, *p* < 0.001)**
51	da Silva et al. [29]	CTL4i + PD1i **140**	CR **31** PR **62** SD **8** PD **39**	LDH Liver metastasis	OS PFS	CTL4i + PD1i (MVA-OS) Liver metastasis (yes vs. no) **(HR 4.03, 95% CI: 1.32 to 12.29, *p* = 0.0143)** CTL4i + PD1i (MVA-PFS) LDH (elevated vs. normal) **(HR 2.99, 95% CI: 1.53 to 5.84, *p* = 0.0013)**
52	Hamid et al. [59]	PD-L1i **45**	CR **4** PR **9** SD **11** PD **18** UN **1**	TMB	OS PFS	PD-L1i (UVA-PFS) TMB (low vs. high) **(HR 0.13, 95% CI, 0.03 to 0.47)**
53	Massi et al. [65]	BRAF + MEKi **64**	-	PD-L1 levels	PFS	BRAFi (MVA-PFS) PD-L1 levels (high vs. low) **(HR 1.99, 95% CI: 1.07 to 3.71, *p* < 0.029)**
54	Cowey et al. [61]	ICIs + Targeted + Other **484** PD1i **179** CTL4i **128** CTL4i/PD1i **20** BRAF + MEKi **96** BRAF/MEKi **31** Other **30**	-	BM ECOG	OS PFS	ICIs + Targeted + Other (MVA-OS) BM (yes vs. no) **(HR 2.89, 95% CI: 1.80 to 4.63, *p* < 0.001)** ICIs + Targeted + Other (MVA-PFS) BM (yes vs. no) **(HR 2.89, 95% CI: 1.8 to 4.63, *p* < 0.001)** ICIs + Targeted + Other (UVA-OS) ECOG (≥2 vs. <2) **(HR 1.92, 95% CI: 1.19 to 3.12, *p* < 0.008)**
55	Ascierto et al. [23]	PD1i **71**		LDH	OS PFS	PD1i (MVA-OS) LDH (elevated vs. normal) **(HR 2.60, 95% CI: 1.24 to 5.43, *p* = 0.011)** PD1i (MVA-PFS) LDH (elevated vs. normal) **(HR 2.26, 95% CI: 1.10 to 4.66, *p* = 0.027)**
56	Richtig et al. [50]	CTL4i **76**	-	BMI	OS	CTL4i (UVA-OS) BMI (≥25 kg/m vs. <25 kg/m^2^) **(HR 0.55, 95% Cl: 0.3 to 1.02, *p* = 0.05)**
57	Taquin et al. [54]	PD1i **91** CTL4i **19**	PD1i CR **11** PR **22** SD **19** PD **39**	BM	OS	PD1i (MVA-OS) BM (yes vs. no) **(HR 2.08, 95% CI: 1.11 to 3.90, *p* = 0.022)**
58	Amini-Adle et al. [22]	PD1i/PD1i **74**	CR + PR **20** SD **9** PD **45**	BRAF	OS	PD1i or PD1i (UVA-OS) BRAF (mutant vs. wild type) **(HR 2.5, 95%CI, 1.3 to 4.5, *p* = 0.005)**
59	Amaria et al. [31]	BRAF/MEKi **11**	CR **2** PR **8** SD **1**	ECOG	OS	BRAF/MEKi (MVA-OS) ECOG (≥2 vs. <2) **(HR 2.35, 95% CI: 1.60 to 3.45, *p* < 0.001)**
60	Kraft et al. [63]	CTLA4i/CT/RT/NA **66**	-	PD-L1 levels	PFS	CTLA4i/CT/RT/NA (MVA-PFS) PD-L1 levels (high vs. low) **(HR 3. 82, 95% CI: 1.18 to 12.4, *p* < 0.026)**
61	Valpione et al. [64]	PD-L1i **41** PD-L1i + CTLA4i **19**	-	Circulating tumor DNA (ctDNA)	OS	PD-L1i/PD-L1i + CTLA4i/BRFAi-MEKi (MVA-OS) ctDNA (high vs. low) **(HR 2.22, 95% CI: 1.18 to 5.55, *p* = 0.012)**
62	Massi et al. [66]	BRAFi **80**	-	PD-L1 levels	PFS	BRAFi (MVA-PFS) PD-L1 levels (high vs. low) **(HR 4.28, 95% CI: 2.10 to 8.72, *p* < 0.001)**
63	McQuade et al. [67]	CTL4i **226** PD-L1i **373**		BMI	OS	PD-L1i (MVA-OS) BMI (≥25 kg/m^2^ vs. <25 kg/m^2^) **(HR 0.78, 95% CI: 0.52 to 1.17, *p* < 0.001)** CTLA4i (MVA-OS) BMI (≥25 kg/m^2^ vs. <25 kg/m^2^) **(HR 0.7, 95% CI: 0.48 to 1.03, *p* < 0.001)**

HRs typed in bold for reader convenience.

## Data Availability

More data and results are available on demand.

## Supplementary Materials

The following supporting information can be downloaded at: https://www.mdpi.com/article/10.3390/jcm15062145/s1, Table S1. Full electronic search strategy for MEDLINE (via PubMed). Table S2. Full electronic search strategy for Web of Science. Table S3. Full electronic search strategy for the Cochrane Central Register of Controlled Trials (CENTRAL). Table S4. Newcastle–Ottawa Scale (NOS) quality assessment for all included studies. Table S5. PRISMA 2020 checklist.

## Abbreviations

**Abbreviations**	**Meaning**
AJCC	American Joint Committee on Cancer
BMI	Body Mass Index
BRAF	B-Raf proto-oncogene
CENTRAL	Cochrane Central Register of Controlled Trials
CI	Confidence Interval
CTLA-4	Cytotoxic T-Lymphocyte-Associated Antigen 4
ctDNA	Circulating Tumor DNA
ECOG	Eastern Cooperative Oncology Group
FDA	U.S. Food and Drug Administration
HR	Hazard Ratio
ICIs	Immune Checkpoint Inhibitors
IFN	Interferon
irAEs	Immune-Related Adverse Events
LDH	Lactate Dehydrogenase
MOOSE	Meta-Analysis of Observational Studies in Epidemiology
MVA	Multivariate Analysis
NOS	Newcastle–Ottawa Scale
NRAS	Neuroblastoma RAS Viral Oncogene Homolog
OS	Overall Survival
PD-L1	Programmed Death-Ligand 1
PFS	Progression-Free Survival
PRISMA	Preferred Reporting Items for Systematic Reviews and Meta-Analyses
SLN	Sentinel Lymph Node
TCR	T-Cell Receptor
TILs	Tumor-Infiltrating Lymphocytes
TMB	Tumor Mutational Burden
TT	Targeted Therapy
UVA	Univariate Analysis
