# Supplementary material for: Clinical, Dermatoscopic, Histological and Molecular Prognostic and Predictive Factors of Metastatic Melanoma Response to Immunotherapy: A Systematic Review and Drug Class Meta-Analysis
Source: J Clin Med. 2026 Mar 11;15(6):2145. doi: 10.3390/jcm15062145 (PMC13026811; doi:10.3390/jcm15062145)
Supplement: Supplementary file 1 [file jcm-15-02145-s001.zip › jcm-4099446-supplementary.pdf]

Table S1. Search strategy for Web of Science.

No.	Criteria	Strings	Hits
Melanoma-related terms			
1	Melanoma – free text	TS=(melanoma*)	180,000
Immunotherapy-related terms			
2	Immunotherapy – general terms	TS=(immunotherap* OR immuno-oncology OR "immune modulation")	105,000
3	Immune checkpoint inhibitors	TS=("immune checkpoint" OR "immune checkpoint inhibitor*" OR ICI)	78,000
4	Molecular targets	TS=("PD-1" OR "PD-L1" OR "CTLA-4")	84,000
5	Drug names	TS=(nivolumab OR pembrolizumab OR ipilimumab)	63,000
6	Mechanistic terms	TS=("anti-PD-1" OR "anti-CTLA-4")	41,000
7	Combined immunotherapy filter	#2 OR #3 OR #4 OR #5 OR #6	260,000
Epidemiological study terms			
8	Study design – named terms	TS=("epidemiologic study" OR "cohort study" OR "case control study" OR "longitudinal study")	620,000
9	Study design – broader scope	TS=("prospective study" OR "retrospective study" OR "observational study")	580,000
10	Combined epidemiology filter	#8 OR #9	1,200,000
Stepwise combination			
11	Melanoma AND Immunotherapy	#1 AND #7	55,000
12	Melanoma AND Immunotherapy AND Epidemiology	#11 AND #10	7,800
Exclusions and limits			
13	Exclude non-original literature	NOT TS=("case report" OR editorial OR letter)	—
14	Human studies only (subject area filter)	Refined by Web of Science Categories: (Oncology OR Immunology OR Public Environmental Occupational Health)	—
15	Time & language limits	Refined by: Year=2018–2025; Language=English	—
16	Final result set	#12 AND #13 AND #14 AND #15	1218

Table S2. Search strategy for Web of Science.

No.	Criteria	Strings	Hits
Melanoma-related terms			
1	Melanoma (MeSH)	"Melanoma"[MeSH Terms]	155,412
2	Melanoma (free-text)	melanoma*[tiab]	182,308
3	Combined melanoma filter	#1 OR #2	189,774
Immunotherapy-related terms			
4	Immunotherapy (MeSH)	"Immunotherapy"[MeSH Terms]	57,204
5	Immune checkpoint terms	"immune checkpoint"[tiab] OR "immune checkpoint inhibitor*"[tiab] OR ICI[tiab]	41,782
6	Molecular targets	"PD-1"[tiab] OR "PD-L1"[tiab] OR "CTLA-4"[tiab]	64,119
7	Drugs	nivolumab[tiab] OR pembrolizumab[tiab] OR ipilimumab[tiab]	54,773
8	Mechanism keywords	"anti-PD-1"[tiab] OR "anti-CTLA-4"[tiab]	28,631
9	Immuno-oncology	immuno-oncology[tiab]	13,980
10	Combined immunotherapy filter	#4 OR #5 OR #6 OR #7 OR #8 OR #9	162,051
Epidemiology-related terms			
11	Epidemiologic studies (MeSH)	"Epidemiologic Studies"[MeSH Terms] OR "Cohort Studies"[MeSH Terms] OR "Case-Control Studies"[MeSH Terms] OR "Prospective Studies"[MeSH Terms] OR "Retrospective Studies"[MeSH Terms] OR "Observational Studies"[MeSH Terms]	647,201
12	Epidemiologic studies (free-text)	"epidemiologic study"[tiab] OR "observational study"[tiab] OR "cohort study"[tiab] OR "case control study"[tiab] OR "longitudinal study"[tiab] OR "prospective study"[tiab] OR "retrospective study"[tiab]	819,083
13	Combined epidemiology filter	#11 OR #12	1,049,911
Combinations			
14	Melanoma AND Immunotherapy	#3 AND #10	35,871
15	+ Epidemiologic studies	#14 AND #13	6,211
Exclusion & Limits			
16	Exclude non-original publications	NOT (editorial[pt] OR letter[pt] OR "case reports"[pt])	—
17	Humans only	humans[MeSH Terms]	—
18	Time & language filter	"2018/01/01"[PDAT] : "2025/12/31"[PDAT] AND English[lang]	—
19	Final result set	#15 AND #16 AND #17 AND #18	1432

Table S3. Search strategy for Cochrane Library.

No.	Criteria	Strings	Hits
1	Melanoma filter	(melanoma*): title abstract keyword	7,089
2	Immunotherapy filter	(immunotherap* OR "immune checkpoint" OR ICI OR PD-1 OR CTLA-4 OR nivolumab OR pembrolizumab OR ipilimumab): title abstract keyword	25,528
3	Epidemiology filter	(cohort OR "case-control" OR observational OR retrospective OR prospective OR longitudinal): title abstract keyword	32,023
4	Melanoma AND Immunotherapy	1 AND 2	2,565
5	Melanoma AND Immunotherapy AND Epidemiology	4 AND 3	976
6	Limits	Limit 5 to 2018 to current, English, Humans	570

Table S4. Newcastle – Ottawa Scale for Risk of Bias assessment.

Study	Year	Selection				Comparability	Outcome			Total
		1	2	3	4		1	2	3	
Majidova et al.	2025	b	a	b	a	a,b	a	a	d	8
Martinez-Recio et al.	2025	b	a	d	a	a	a	d	a	7
Pedersen et al.	2025	a	a	a	b	a	a	a	a	8
Zhang et al.	2025	b	a	b	a	a,b	a	a	a	9
Jansen et al.	2025	b	a	a	a	a,b	b	a	d	8
Derks et al.	2025	b	a	d	a	a,b	b	a	d	7
Verkhovskaia et al.	2025	b	a	a	a	b,a	b	a	b	9
Kott et al.	2025	b	a	a	a	a,b	b	a	b	9
Silasi et al.	2024	a	a	d	a	a,b	a	a	d	7
Mandala et al.	2024	a	a	b	a	a,a	a	b	d	8
Chen et al.	2024	a	a	b	d	a	a	a	d	7
Kopecký et al.	2024	a	a	b	a	a	a	a	b	8
Sun et al.	2024	b	a	a	a	a,b	b	a	d	8
Pires da Silva et al.	2024	b	a	a	b	a	b	a	d	6
Falcone et al.	2024	b	a	a	a	a	b	a	b	8
Jan et al.	2024	b	a	a	a	a,b	b	a	d	8
Zhao et al.	2024	b	a	a	a	a,b	b	a	b	9
Long et al.	2024	b	a	a	a	a,b	b	a	b	9
Marchisio et al.	2024	b	a	d	a	a	b	a	d	7
Roccuzzo et al.	2024	b	a	d	a	a,b	b	a	d	7
Rozendorn et al.	2024	b	a	d	a	a,b	b	a	b	8
Roccuzzo et al.	2024	b	a	a	a	a,b	b	a	d	8
Tang et al,	2024	b	a	a	a	a	b	a	b	8
Cybulska-Stopa et al.	2023	a	a	a	a	a	a	a	a	9
Bloem et al.	2023	b	a	d	a	a	b	a	d	6
Lo et al.	2023	b	a	a	a	a,b	b	a	b	9
Kartolo et al.	2023	b	a	a	a	a,b	b	a	b	9
Internò et al.	2023	b	a	a	b	a	b	a	b	8
Rousset et al.	2023	b	a	d	a	a	b	a	d	7
Haist et al.	2023	b	a	b	a	a,b	a	a	d	8
Namikawa et al.	2023	a	a	b	a	a,b	a	a	d	8
Afrăsânie et al.	2023	b	a	a	b	a,b	b	a	a	8

Franklin et al.	2023	b	a	b	a	a,b	b	a	d	8
Carvajal et al.	2022	b	a	d	a	a,b	b	a	d	7
Vavolizza.	2022	b	a	a	a	a,b	b	a	b	9
Carvajal et al.	2022	b	a	a	a	a,b	b	a	b	9
Li et al.	2022	b	a	a	b	a	b	a	b	8
Robert et al.	2022	b	a	d	a	a	b	a	d	7
Tarhini et al.	2022	b	a	a	a	a,b	b	a	d	8
Kim et al.	2022	b	a	a	a	A,b	b	a	b	9
Nardin et al.	2022	b	a	d	a	a,b	b	a	d	7
Yamada et al.	2021	b	a	a	a	a	b	a	d	7
Hodi et al.	2021	b	a	a	a	a,b	b	a	b	9
Zhou et al.	2021	b	a	a	a	a,b	b	a	d	8
Yamazaki et al.	2021	b	a	a	a	a	b	a	d	7
Tarhini et al.	2021	b	a	a	a	a,b	b	a	b	9
Guida et al.	2021	b	a	a	a	a,b	b	a	d	8
Huber et al.	2021	b	a	a	a	a,b	b	a	d	8
Maurer et al.	2020	b	a	a	b	a.b	b	b	d	6
Cowey et al.	2020	b	a	d	a	a,b	b	a	d	7
Săftescu et al.	2020	b	a	d	a	a,b	b	b	d	7
Puzanov et al.	2020	b	a	a	a	a,b	b	a	d	8
Yamazaki et al.	2020	b	a	a	a	a,b	b	a	b	8
Ratnayake et al.	2020	b	a	a	a	a	b	a	b	8
Lepletier et al.	2020	b	a	a	a	a,b	b	a	b	9
Pires da Silva.	2019	b	a	a	a	a	b	a	d	7
Pinato et al.	2019	b	a	d	a	a,b	b	b	b	7
Hamid et al.	2019	b	a	d	a	a,b	b	a	d	7
Cowey et al.	2019	b	a	a	a	a,b	b	a	d	8
Rossi et al.	2019	b	a	a	a	a,b	b	a	b	9
Ascierto et al.	2019	b	a	a	a	a	b	a	d	7
Gibney et al.	2019	b	a	a	a	a,b	b	a	b	9
Fässler et al.	2019	b	a	a	a	a,b	b	b	d	7
Luke et al.	2019	b	a	a	b	a,b	b	a	b	8
Richtig et al.	2018	b	a	a	a	a	b	a	b	8
Taquin et al.	2018	b	a	a	a	a	b	a	b	8
Amini-Adle et al.	2018	b	a	a	b	a,b	b	a	d	7

Amaria et al.	2018	b	a	d	a	a	b	a	b	7
Haag et al.	2018	b	a	a	a	a,b	b	a	d	8

Table S5. PRISMA 2020 checklist.

Section and Topic	Item #	Checklist item	Location where item is reported
**TITLE**			
Title	1	Identify the report as a systematic review.	1
**ABSTRACT**			
Abstract	2	See the PRISMA 2020 for Abstracts checklist.	1
**INTRODUCTION**			
Rationale	3	Describe the rationale for the review in the context of existing knowledge.	1-2
Objectives	4	Provide an explicit statement of the objective(s) or question(s) the review addresses.	1-2
**METHODS**			
Eligibility criteria	5	Specify the inclusion and exclusion criteria for the review and how studies were grouped for the syntheses.	4-5
Information sources	6	Specify all databases, registers, websites, organisations, reference lists and other sources searched or consulted to identify studies. Specify the date when each source was last searched or consulted.	4-5
Search strategy	7	Present the full search strategies for all databases, registers and websites, including any filters and limits used.	4
Selection process	8	Specify the methods used to decide whether a study met the inclusion criteria of the review, including how many reviewers screened each record and each report retrieved, whether they worked independently, and if applicable, details of automation tools used in the process.	4-5
Data collection process	9	Specify the methods used to collect data from reports, including how many reviewers collected data from each report, whether they worked independently, any processes for obtaining or confirming data from study investigators, and if applicable, details of automation tools used in the process.	4-5
Data items	10a	List and define all outcomes for which data were sought. Specify whether all results that were compatible with each outcome domain in each study were sought (e.g. for all measures, time points, analyses), and if not, the methods used to decide which results to collect.	6
	10b	List and define all other variables for which data were sought (e.g. participant and intervention characteristics, funding sources). Describe any assumptions made about any missing or unclear information.	6
Study risk of bias assessment	11	Specify the methods used to assess risk of bias in the included studies, including details of the tool(s) used, how many reviewers assessed each study and whether they worked independently, and if applicable, details of automation tools used in the process.	6
Effect measures	12	Specify for each outcome the effect measure(s) (e.g. risk ratio, mean difference) used in the synthesis or presentation of results.	6-7
Synthesis methods	13a	Describe the processes used to decide which studies were eligible for each synthesis (e.g. tabulating the study intervention characteristics and comparing against the planned groups for each synthesis (item #5)).	6-7
	13b	Describe any methods required to prepare the data for presentation or synthesis, such as handling of missing summary statistics, or data conversions.	6-7
	13c	Describe any methods used to tabulate or visually display results of individual studies and syntheses.	6-7
	13d	Describe any methods used to synthesize results and provide a	6-7

Section and Topic	Item #	Checklist item	Location where item is reported
		rationale for the choice(s). If meta-analysis was performed, describe the model(s), method(s) to identify the presence and extent of statistical heterogeneity, and software package(s) used.	
	13e	Describe any methods used to explore possible causes of heterogeneity among study results (e.g. subgroup analysis, meta-regression).	6-7
	13f	Describe any sensitivity analyses conducted to assess robustness of the synthesized results.	6-7
Reporting bias assessment	14	Describe any methods used to assess risk of bias due to missing results in a synthesis (arising from reporting biases).	6-7
Certainty assessment	15	Describe any methods used to assess certainty (or confidence) in the body of evidence for an outcome.	6-7
**RESULTS**			
Study selection	16a	Describe the results of the search and selection process, from the number of records identified in the search to the number of studies included in the review, ideally using a flow diagram.	7-34
	16b	Cite studies that might appear to meet the inclusion criteria, but which were excluded, and explain why they were excluded.	7-34
Study characteristics	17	Cite each included study and present its characteristics.	43-56
Risk of bias in studies	18	Present assessments of risk of bias for each included study.	7-34
Results of individual studies	19	For all outcomes, present, for each study: (a) summary statistics for each group (where appropriate) and (b) an effect estimate and its precision (e.g. confidence/credible interval), ideally using structured tables or plots.	7-34
Results of syntheses	20a	For each synthesis, briefly summarise the characteristics and risk of bias among contributing studies.	7-34
	20b	Present results of all statistical syntheses conducted. If meta-analysis was done, present for each the summary estimate and its precision (e.g. confidence/credible interval) and measures of statistical heterogeneity. If comparing groups, describe the direction of the effect.	7-34
	20c	Present results of all investigations of possible causes of heterogeneity among study results.	7-34
	20d	Present results of all sensitivity analyses conducted to assess the robustness of the synthesized results.	7-34
Reporting biases	21	Present assessments of risk of bias due to missing results (arising from reporting biases) for each synthesis assessed.	7-34
Certainty of evidence	22	Present assessments of certainty (or confidence) in the body of evidence for each outcome assessed.	7-34
**DISCUSSION**			
Discussion	23a	Provide a general interpretation of the results in the context of other evidence.	34-42
	23b	Discuss any limitations of the evidence included in the review.	42
	23c	Discuss any limitations of the review processes used.	42
	23d	Discuss implications of the results for practice, policy, and future research.	43
**OTHER INFORMATION**			
Registration and protocol	24a	Provide registration information for the review, including register name and registration number, or state that the review was not registered.	43
	24b	Indicate where the review protocol can be accessed, or state that a protocol was not prepared.	43
	24c	Describe and explain any amendments to information provided at registration or in the protocol.	43
Support	25	Describe sources of financial or non-financial support for the review, and the role of the funders or sponsors in the review.	43

Section and Topic	Item #	Checklist item	Location where item is reported
Competing interests	26	Declare any competing interests of review authors.	43
Availability of data, code and other materials	27	Report which of the following are publicly available and where they can be found: template data collection forms; data extracted from included studies; data used for all analyses; analytic code; any other materials used in the review.	43
